# Highly Integrated Multiplexing and Buffering Electronics for Large Aperture Ultrasonic Arrays

**DOI:** 10.34133/2022/9870386

**Published:** 2022-06-30

**Authors:** Robert Wodnicki, Haochen Kang, Di Li, Douglas N. Stephens, Hayong Jung, Yizhe Sun, Ruimin Chen, Lai-Ming Jiang, Nestor E. Cabrera-Munoz, Josquin Foiret, Qifa Zhou, Katherine W. Ferrara

**Affiliations:** 1 Department of Biomedical Engineering, University of Southern California, Los Angeles, CA, USA; 2 Department of Biomedical Engineering, University of California, Davis, Davis, CA, USA; 3 Molecular Imaging Program at Stanford University, Stanford, CA, USA; 4 USC Roski Eye Institute, University of Southern California, Los Angeles, CA, USA

## Abstract

Large aperture ultrasonic arrays can be implemented by tiling together multiple pretested modules of high-density acoustic arrays with closely integrated multiplexing and buffering electronics to form a larger aperture with high yield. These modular arrays can be used to implement large 1.75D array apertures capable of focusing in elevation for uniform slice thickness along the axial direction which can improve image contrast. An important goal for large array tiling is obtaining high yield and sensitivity while reducing extraneous image artifacts. We have been developing tileable acoustic-electric modules for the implementation of large array apertures utilizing Application Specific Integrated Circuits (ASICs) implemented using 0.35 ***μ***m high voltage (50 V) CMOS. Multiple generations of ASICs have been designed and tested. The ASICs were integrated with high-density transducer arrays for acoustic testing and imaging. The modules were further interfaced to a Verasonics Vantage imaging system and were used to image industry standard ultrasound phantoms. The first-generation modules comprise ASICs with both multiplexing and buffering electronics on-chip and have demonstrated a switching artifact which was visible in the images. A second-generation ASIC design incorporates low switching injection circuits which effectively mitigate the artifacts observed with the first-generation devices. Here, we present the architecture of the two ASIC designs and module types as well imaging results that demonstrate reduction in switching artifacts for the second-generation devices.

## 1. Introduction

Ultrasonic transducer arrays are used extensively in modern medical practice. Linear arrays, composed of a row of individual elements, have been broadly applied in the diagnosis and staging of malignancies in the liver, kidney, breast, and thyroid [1, 2]. Phased arrays, in which the elements emit a focused beam that is steered, have found application in cardiology for diagnosis of structural abnormalities in the heart muscle and valves [3]. Curvilinear arrays combine aspects of linear and phased arrays and are widely used for abdominal and fetal imaging [1]. The majority of these transducer arrays have a one-to-one mapping between the ultrasound system channels and the individual array elements. Newer probes incorporate electronic multiplexing [4–7] or analog micro-beamforming operations [8–10] in the probe itself to allow for a larger number of elements to be used with the existing 128 or 256 channel digital beamforming ultrasound systems. In particular, 2D and 1.75D multiplexed arrays have shown promise for improved contrast which is of particular importance for detection and differentiation of cancerous lesions [4, 11].

**Figure 1:**
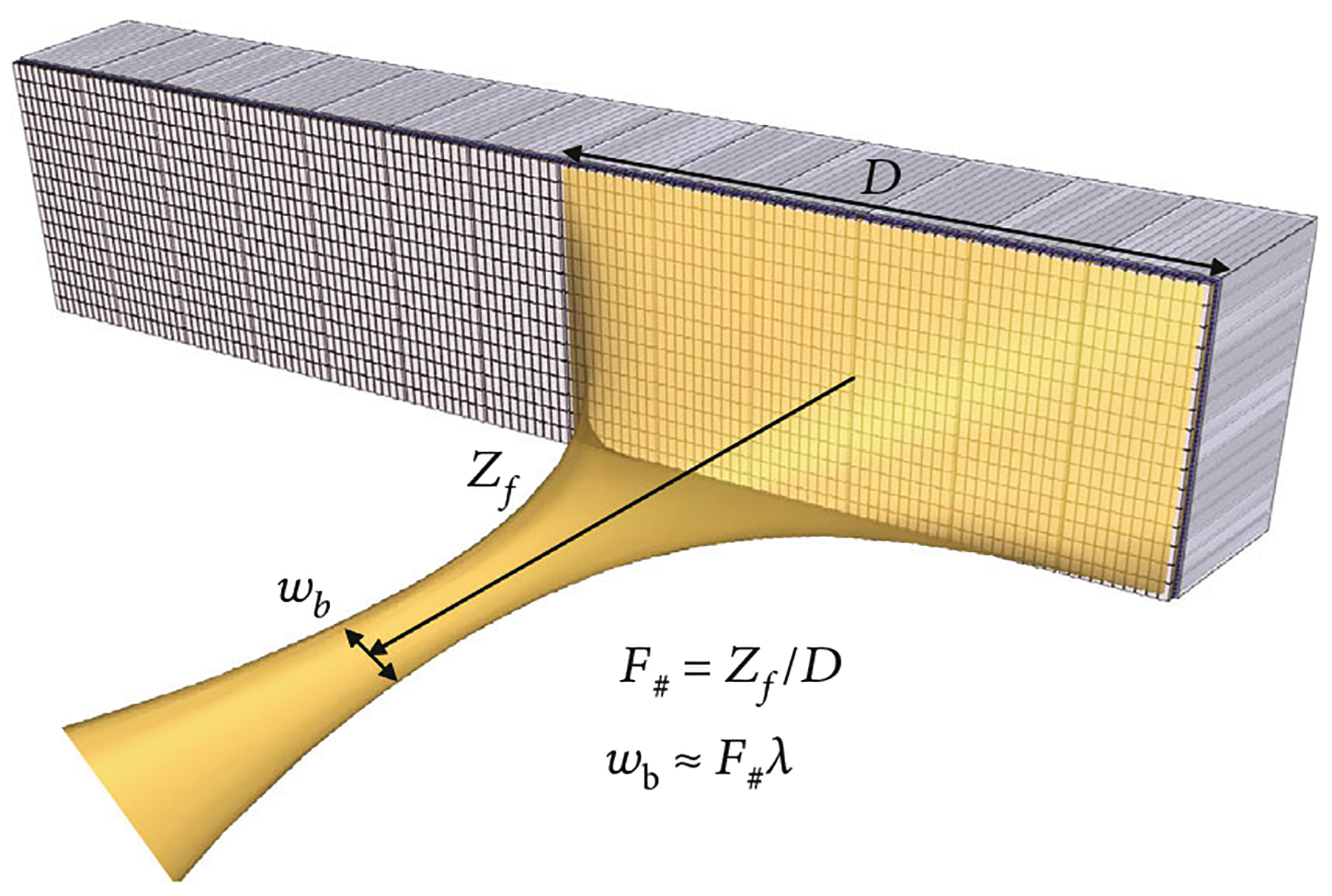
Illustration of a large aperture ultrasonic array which has benefits for imaging of deep lesions for cancer diagnosis and screening. These include improved contrast due to electronic focus in elevation and improved lateral resolution due to a reduced F#.

Large apertures of 1.75D and 2D arrays of elements with beamforming in an extended elevational dimension are advantageous due to their increased penetration and reduced imaging slice thickness which in turn improves contrast at depth (Figure 1). These arrays are highly focused in the elevational dimension to greatly improve the thickness uniformity of the imaged plane and thereby extend the depth of focus of the receive beam. The focal width is reduced in the elevational plane for improved contrast to noise (CNR) of in-plane features which is critical for correctly resolving fluid-filled cysts and is important for differential diagnosis of cancer [11, 12]. In addition, large apertures with spatial compounding in the lateral dimension are beneficial for improving lateral resolution [13]. A large aperture has a low F#, and since the lateral resolution varies directly with F# [2], finer resolution can be obtained at lower frequency providing greater penetration depths into the body (Figure 1).

There are a number of advanced applications which are the subject of recent research that will benefit from fully beamformed apertures that are larger than what are currently available in commercial systems. These applications include automated breast ultrasound [14–17], plane wave imaging with large matrix arrays for high-speed acquisition of volumes enabling 3D mapping of stiffness and blood flow in cardiology applications [18], as well as high intensity focused ultrasound (HIFU) for therapeutic treatment of cancer [19]. Another important potential application for a large array is screening and surveillance for hepatocellular carcinoma (HCC) [13, 16]. The high risk of mortality of HCC provides significant incentive to diagnose when it can still be treated [20–22]. B-mode ultrasound is an important screening modality in this regard [23] due to the fact that patients with cirrhotic liver are typically followed at very close intervals (3–6 months) and therefore an inexpensive procedure is preferred. The prevalence of NASH-related cirrhosis is becoming an increasingly important disease etiology for HCC in North America [24], and therefore the ability to correctly detect and identify HCC lesions in fibrotic liver for the obese patient population is of increasing importance. Detection of deep lesions in the fibrotic liver in the presence of significant aberrating fat layers is challenging and requires improvements in penetration and contrast which can be ameliorated through the use of large apertures [13].

A large aperture array with increased lateral and elevational dimensions requires means for processing many more 2D and 1.75D array elements than available system channels. This presents a bottleneck in terms of signal processing capability which must be resolved before a large aperture array can be constructed. Multiplexed (“muxed”) probes, integrating commercially available high voltage multiplexing electronics either on the system side of the cable or in the probe handle, were introduced to expand the number of elements beyond the limitations of existing systems, increasing the number of elements from 128 system channels to 192 or more elements to enlarge the field of view [1, 2] and allow for the implementation of expanding aperture in elevation for improved contrast to noise ratio (CNR) [4, 11, 25]. More recently, analog micro-beamforming [26, 27] adjacent to [5] or immediately behind [8–10] the 2D transducer array has also been used to implement large arrays of transducer elements. Finally, the concept of multiplexing has been expanded to create large arrays with dense reconfigurable switching circuits immediately behind the array [7, 28].

Large area ultrasonic arrays with locally integrated multiplexing networks can be implemented by tiling multiple pretested modules together to form a larger aperture with high yield [16]. These modular arrays can be used to implement large 1.75D array apertures capable of focusing in elevation for uniform slice thickness along the axial direction which can improve image contrast. An important goal for large array tiling is obtaining high yield and sensitivity while reducing extraneous image artifacts. We have been developing tileable modules for implementation of large array apertures utilizing high voltage Application Specific Integrated Circuits (ASICs) implemented using 0.35 *μ*m high voltage (50 V) CMOS [29]. Multiple generations of ASICs have been designed and tested [30, 31]. The ASICs were integrated with high-density 1.75D transducer arrays implemented using wide-bandwidth 1–3 composites of PIN-PMN-PT material [32] and a 3D printed interposer backing for high-density interface [33]. The individual modules implement a 6 × 20 array of transducer elements at 600 *μ*m (azimuth) × 1600 *μ*m (elevation pitch with a nominal center frequency of 3.5 MHz. The modules were further interfaced to a Verasonics Vantage imaging system and were used to image industry standard ultrasound phantoms. The first-generation modules comprise ASICs with both multiplexing and buffering electronics on-chip and have demonstrated a switching artifact which was visible in the images. A second-generation ASIC design incorporates low switching injection circuits which effectively mitigate the artifacts observed with the first-generation devices.

Important aspects of large arrays with small 2D and 1.75D array elements include optimization of buffering to create improved matching of the high impedance elements to external load capacitances (e.g., probe cable and system electronics) to prevent loss of signal and thereby improve penetration and contrast. As opposed to historical electronics for ultrasound which assumed large transducer elements with low electrical impedance and 50 *Ω* matched systems, the high impedance of dense 2D array elements make it possible to utilize multiplexing switches which also have high impedance (e.g., hundreds of ohms). This leads to considerable savings in ASIC implementation area and a reduction in matrix loading and injection artifacts which the designs presented here take advantage of.

Here, we compare and contrast the architecture of the two ASICs as well as imaging results that demonstrate reduction in switching artifacts for the second-generation devices. We have evaluated the two different module architectures with imaging and detailed electrical performance measurements and provide a comparison of these parameters with industry standard multiplexing and buffering devices.

## 2. Methods

To implement large area arrays, we have developed multiple generations of dense multiplexing networks based on a modular approach for high yield [30, 32, 33]. Low yield of the elements can affect the imaging performance [34, 35] and is especially critical when using highly integrated ASIC devices in the probe handle since the loss of a single ASIC can take out a big part of the active aperture. The modular approach allows for testing of individual parts of the array before assembly which can thereby improve the overall yield for a large array aperture implementation [36, 37]. The modules include ASICs with closely coupled transducer array stacks on a custom high-density printed circuit board [32]. We implemented two successive generations of ASICs with the first generation [30] benefiting from locally integrated buffer amplifiers, and the second generation benefiting from reduced imaging artifacts due to an improved switch architecture that reduces generated spurious glitches [31].

**Figure 2:**
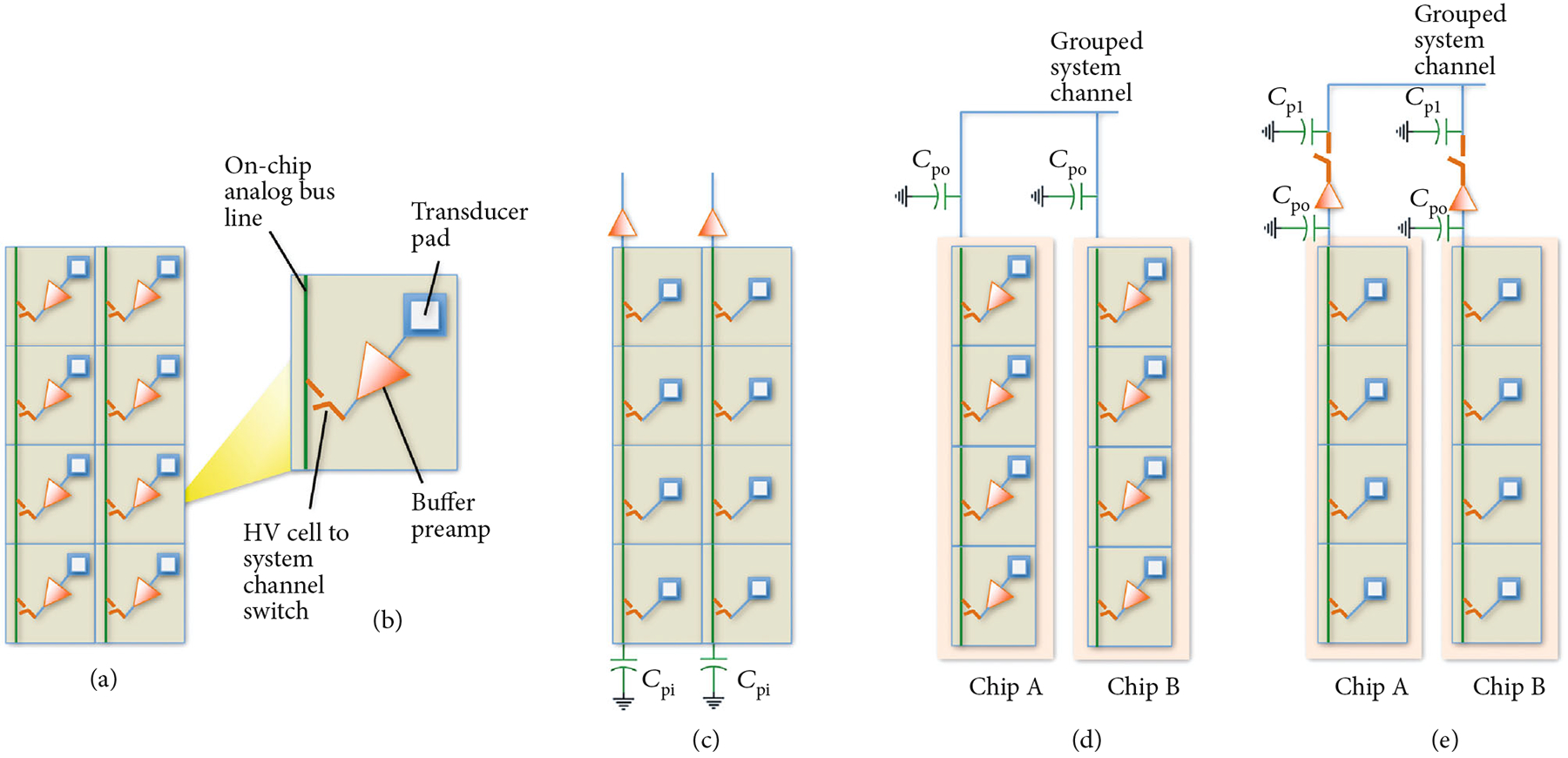
Illustration of different multiplexing and buffering architectures and parasitic effects. (a) First-generation ASICs with a single buffer and high voltage select switch (red); each buffer has a high voltage transmit/receive switch (not shown) that effectively makes this path bidirectional, (b) single-unit cell, (c) second-generation ASIC modules with single buffers (and T/R switch, not shown) off-chip at top of each column, (d) effects of parasitics when multiple ASICs with on-chip buffers are ganged together, and (e) multiple ASICs with off-chip buffers.

Figure 2 illustrates and compares the basic switching architectures for the first- and second-generation ASIC devices and will be used to explain important general design considerations for such implementations. Figure 2(a) illustrates the architecture of the switching matrix implemented by the first-generation ASICs. The switching ASICs are organized in arrays of unit cells (Figure 2(b)) where each unit cell is interfaced directly to a single unique transducer element within the large array. One or more analog bus lines (vertical green lines in Figure 2) are distributed in each column of the ASIC unit cell array, and these are in turn connected to the probe cable which connects back to a unique system channel in the beamformer. In ultrasonic systems, high voltage transmission signals (20 V–200 V) are required to improve penetration of the ultrasound signal deep in the tissue, and this necessitates the use of special-purpose high voltage ASIC fabrication processes [29]. As shown in Figure 2(b), each of the unit cells includes the analog bus line routing as well as high voltage multiplexing switches. The unit cells also have a transducer pad which provides the direct connection to the transducer array elements. In the first-generation ASICs, each unit cell further comprises a locally integrated buffer amplifier with integrated transmit/receive (T/R) switch for a bidirectional transmit/receive path. This buffer itself is a low voltage (<5 V) device which is also protected from the high voltage transmit signals by locally integrated high voltage T/R switches (not shown in the figure). The T/R switch routes the transmit signal around the buffer, thereby creating a bidirectional interface circuit at each element. The second-generation ASIC topology is illustrated in Figure 2(c). Here, the buffer amplifiers have been moved outside of the array and in fact are implemented off-chip using commercially procured ICs (MAX4805, Maxim Integrated).

An important consideration for the design and implementation of dense multiplexing electronics for a large area array is the effects of parasitic capacitances. As illustrated in Figure 2(c), each of the columns of the array has an associated parasitic capacitance C_pi_ which is the aggregate lumped stray capacitance associated with the on-chip routing. These capacitances affect receive sensitivity if their equivalent impedance magnitude is similar to the source impedance of the array elements, effectively loading the transducers. The impedance of the elements increases inversely with their element aperture size, which by design is reduced in a large array with a large number of densely packed 2D or 1.75D array elements. One way to mitigate the effect of the parasitic capacitance and improve the sensitivity is by locally integrating buffer amplifiers at every element such that the loading on every system channel bus line is effectively isolated from the individual elements. This is the architecture choice that was made for the first-generation of ASICs. There exists an important trade-off however in this implementation, and that is that it significantly increases the power dissipation in the array due to the use of a large number of low noise preamplifiers. Therefore, for the second generation of ASICs, we moved to the architecture of Figure 2(c), with buffer amplifiers implemented for each system channel instead of at each element. This reduces the power consumption by a factor of *R*, where *R* is the number of rows in the array (here 8 rows on each ASIC).

A further consideration for buffering is illustrated in Figure 2(d). Here, multiple ASICs are ganged together as would be typical in a large area array with multiplexed scanning in the azimuthal dimension (along the columns). In this case, we need to account for the additional off-chip loading, C_po_, which is due to the signal routing on the printed circuit boards themselves. In general, the on-chip loading is on the order of 1 pF, or less, while loading on the PCB can be between 1 and 10 pF depending on the length of the traces and whether or not connectors are used to interface between modules. For comparison, a typical transducer element in our acoustic stack has a source impedance magnitude near resonance equivalent to a capacitance of 16 pF (4 k*Ω* at 2.5 MHz) when 1.75D array elements are implemented [31]. Therefore, ganging multiple ASICs across multiple module boards requires careful evaluation of the resulting aggregate loading and trading this off against the location and number of preamplifiers to optimize sensitivity, power consumption, and the complexity of the implementation. As a further example, Figure 2(e) illustrates the case where off-chip buffer amplifiers are used. When these are placed very close to each ASIC, C_po_ is small, and instead C_p1_ after the buffer amplifiers may be more important. This additional load capacitance constitutes routing across the PCB and likely also communication through connectors between modules, each of which will add additional load capacitance. It is also important to note in Figure 2(e) that the use of buffers at each system channel now requires additional switches to allow for multiplexing individual columns of elements to the smaller number of system channels for scanning an active window in the azimuthal direction. Buffer amplifiers have been used extensively in 2D array implementations to isolate the loading of the probe cable capacitance, and the system receives impedance from the high impedance individual 2D array elements [9, 10, 38]. For 2D array elements (as opposed to 1.75D) with *λ* pitch in both dimensions, the elements would have higher impedance (e.g., 11 k*Ω* at 2.5 MHz), and for *λ*/2 pitch as required for phased array steering, the impedance would be even higher (>30 k*Ω* at 2.5 MHz). In those cases, buffering at each element (as with the first-generation ASICs in Figure 2(a)) would need to be considered. However, in our current large area array implementations [39, 40], using *λ* pitch 1.75D elements, we have chosen to continue with the second-generation ASICs where the buffering is done off-chip and per system channel.

Switching electronics for ultrasound systems have been available since at least the 1990s in the form of discrete ICs implemented using very high voltage fabrication processes [41]. These devices were designed to operate with large linear array elements and were typically located at the system side of the cable. In this case, they are required to switch not just the capacitance of the large linear array elements themselves (~20–30 pF) but added to that the capacitance of the cable (~100–150 pF). To maintain adequate transmit signal rise times under these conditions, the series resistance of these commercial switches is typically in the range of 10 *Ω*. With electronic switches implemented using MOSFET devices, the on resistance of the switch varies inversely with the size of the devices [42]. Physically very large MOSFETs must be implemented for high voltage operation with low series on resistance. These very large devices also have their own commensurate large parasitic capacitance (~100 pF) and generate switching glitch artifacts (discussed further below) which are due to the large amount of charge needed to create and quench the MOSFET conductive channel when the switches are actuated. In contrast, moving the switching electronics to the probe itself on the other side of the cable and isolated by buffer line drivers makes it possible to match the switch series resistance closer to the transducer impedance magnitude of the small 2D array elements (e.g., 2–6 k*Ω*) which leads to very significant physical size reduction of the switches. This is the design tradeoff that has been implemented in the majority of recent high-density multiplexing electronics [7, 28]. The advantage of this trade-off is that the integration density of the switches themselves is greatly increased, while the parasitic loading and glitch energy are significantly reduced. In our ASIC designs, we have taken advantage of these tradeoffs and will elaborate and compare them in Section 3.

With these considerations in mind, the remainder of this section will detail the design and analysis of the first- and second-generation ASICs. Section 2.1 describes the basic architecture of the devices including the switching circuitry used and the buffering preamplifier. Section 2.1 also examines the design of the second-generation ASICs which have a modified switch architecture that mitigates imaging artifacts observed with the first-generation switch devices. Testing and comparisons between the two devices are provided in Section 3.

### 2.1. First- and Second-Generation ASIC Design and Fabrication.

**Figure 3:**
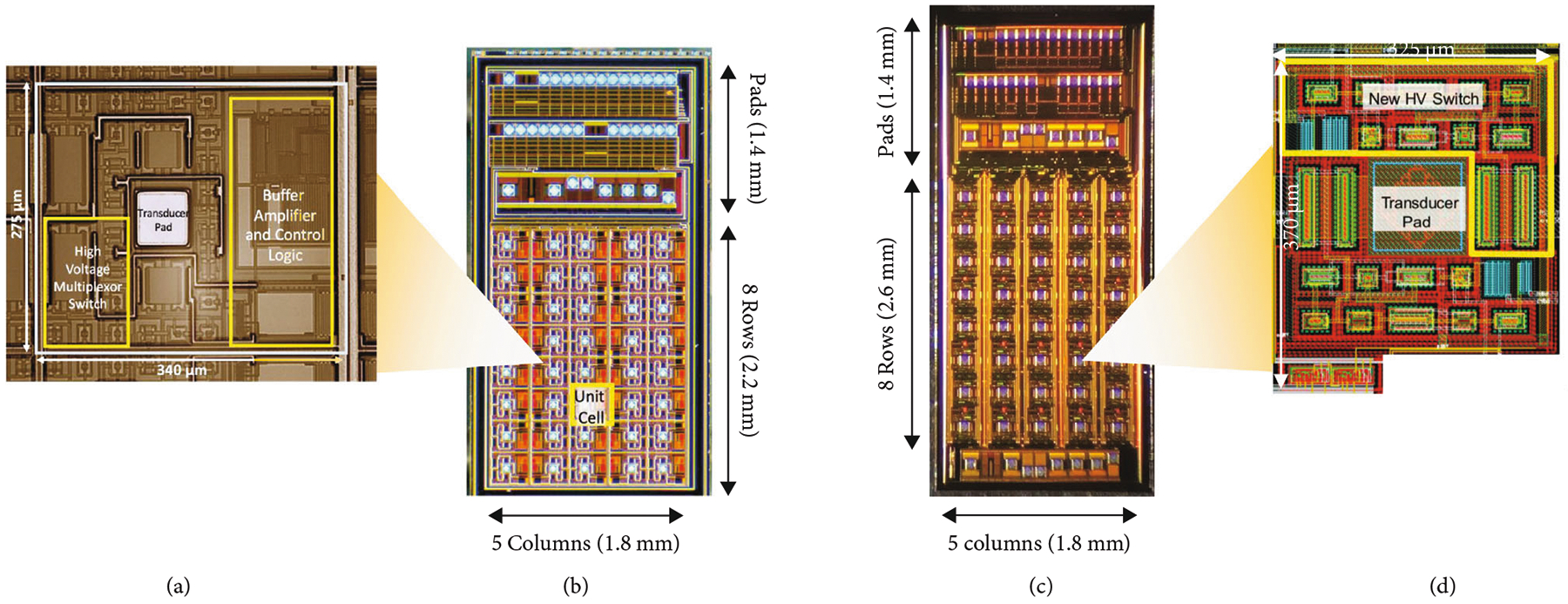
The first- (a, b) and second- (c, d) generation ASICs incorporating multiple mux switches for interfacing to 2D and 1.75D transducer array elements. (a) First-generation ASIC unit cell, (b) complete first-generation device, (c) complete second-generatation ASIC, (d) second-generation ASIC unit cell CAD layout ((b) adapted from [30], (c-d) adapted from [40]).

The first- and second-generation ASICs are designed to multiplex and buffer signals from a 1.75D transducer array, connecting them to the channels of a highly versatile ultrasound system (Verasonics Vantage, Verasonics Inc, Kirkland, WA). As illustrated in Figures 3(a) and 3(b) (adapted from [30]) and Figures 3(c) and 3(d) (adapted from [40]), the first-generation ASIC device comprises an array of 40 interface cells arranged as 5 columns at 340 *μ*m pitch by 8 rows at 275 *μ*m pitch. In all of the arrays described in this paper, for both the ASICs and the acoustic elements, the columns correspond to the azimuthal direction, while the rows correspond to elevation. The purpose of the ASIC is to construct a 1.75D linear array [11] capable of dynamic receive focusing in elevation and limited steering. For a 2D array, access to the large number of elements is the main concern and includes the two issues of connection to the elements and multiplexing to alleviate the interconnect bottleneck. In addition, the small size of the dense acoustic array elements results in a high source impedance which needs to be effectively isolated from cable and system electronics loading to improve receive signal sensitivity. This “matching” of the high impedance elements to low impedance system components is done using buffering amplifiers integrated locally as close as possible to the array elements themselves. The high impedance of the array elements also makes it possible to use high impedance multiplexing switches. These are designed to have 10× lower impedance than the elements (e.g., 100’s of *Ω* vs. k*Ω*) so as to minimize their effect on the transmit and receive signals. These switches still have 10× higher impedance than commercially available switching electronics (e.g., [41]) which yields very significant savings in ASIC area and capacitive loading of these devices.

In the current design, connection to each transducer element is provided by a single aluminum pad located at the center of each unit cell on the ASIC (Figure 3(a)). These are connected to the module PCB using gold wire-bonds. The 2D array routing bottleneck is alleviated by high voltage multiplexing switches located in each cell, with each switch connecting the respective element to an analog bus line running down each column. The switching matrix can be operated to scan a window along the azimuthal direction, and successively multiplex each row of elements in the elevational direction to the 5 system channels. The ASIC layout is designed to be modular in that it can be tiled in the azimuthal direction and mirrored in the elevational direction to create tiled arrays of (5 × *N*) × (8 × 2) elements (where *N* is the number of ASICs in azimuth). For example, a 100 × 16 channel array can be created by tiling 20 chips in azimuth and 2 chips in elevation, for a total of 40 devices. The choice of 5 (rather than 4 or 8) columns was made for an optimized cost of implementation of the test ASICs in the Multi-Project Wafer run.

Outside of the core array of 40 unit cells, the ASICs include an array of input/output (“I/O”) pads (Figures 3(b) and 3(c)). These are laid out in multiple tiers and can be wire-bonded or flip-chip attached to a flex circuit for access to the ultrasound system. The overall size of the first-generation ASIC including the I/O pads is 1800 *μ*m × 3600 *μ*m. The small size of the current design is intended to improve yield and also is constrained by the cost of fabrication.

The second-generation devices (Figures 3(c) and 3(d)) have similar overall matrix array specifications as the first generation; however, they do not have buffer amplifiers at each element. In addition, the second-generation ASICs have reduced charge-injection (described in Section 2.4.4) and also implemented two selection switches for every element. The second bank of selection switches connect to unique channel pads effectively yielding twice as many system channel connections for increased flexibility of access to the elements. The second-generation devices also have a reduced series resistance of their high voltage matrix select switches (330 *Ω* vs. 900 *Ω*). The size of high voltage switches varies from 10× to 20× the size of low voltage switches implemented with the same on resistance, and this leads to a considerable consumption of ASIC area dedicated to these devices. The second-generation ASIC is also slightly taller than the first-generation (4.4 mm vs. 3.6 mm). As discussed in Section 2.2, the ASIC cells themselves are of similar size with different area tradeoffs given their different design. The reason that the second-generation devices are taller overall is due to the use of a second bank of analog channel connection pads located along the bottom of the device. This second set of pads provides unique channel connections for the second bank of switches in each unit cell.

Both ASICs shown in Figure 3 were designed using Cadence Virtuoso for schematic capture. The designed unit cells were simulated using a simple model for the transducers over the design corners. These were then built up to a complete schematic of the 40 unit cells. Layout was done using Cadence, and layout vs. schematic was used to validate the layout relative to the simulated unit cells, and later for the complete top-level design. For both ASICs, the final layout data was fabricated using a 0.35 *μ*m 50 V CMOS fabrication process (AMS H35) through a Multi-Chip Wafer (MPW) service (Europractice/Fraunhofer, Erlangen Germany) [29].

### 2.2. Unit Cell Architecture.

**Figure 4:**
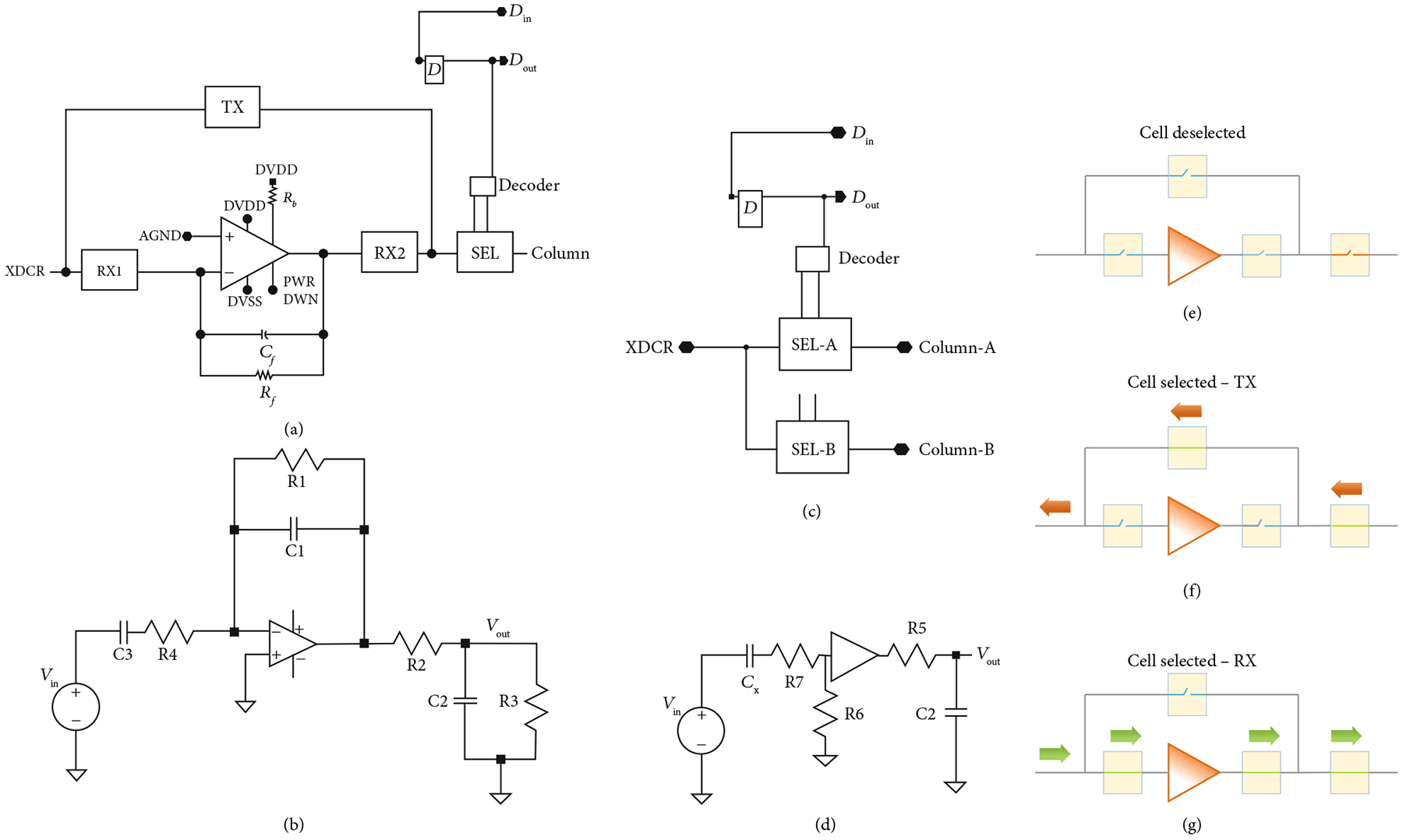
Schematic representation of the ASIC unit cells. (a) First-generation with integrated buffer, illustrating the transmit/receive protection high voltage switches (TX, RX1, and RX2) as well as the matrix multiplexing switch (SEL), and digital data storage D flip-flop connected to DIN, (b) small signal circuit model for (a), (c) second-generation ASIC unit cell without buffer and transmit/receiving circuit, (d) small-signal circuit model for (c), operation of the unit cell of (a) when (e) deselected, (f) selected in transmit mode, and (g) selected in receive mode. The red arrows show the transmit signal path, while the green arrows show the receive signal path.

A schematic of the unit cell circuitry for the first- and second-generation unit cell is illustrated in Figure 4. Each first-generation unit cell (Figure 4(a)) contains a transimpedance buffer along with 4 high voltage switches (TX, RX1, RX2, and SEL). Three of the high voltage switches (TX, RX1, and RX2) make up the transmit/receive switch to protect the amplifier from the transmit signal and also route it around the amplifier. The fourth switch (SEL) is the select switch which is used to multiplex the system channels to the individual elements in the array. Connection to the respective transducer of the unit cell is provided by the interface pad at the connection of the TX and RX1 switches. The second-generation devices (Figure 4(c)) have similar overall matrix composition; however, there is no buffer in each cell, and the select switches have reduced charge-injection (Section 2.4.4).

**Figure 5:**
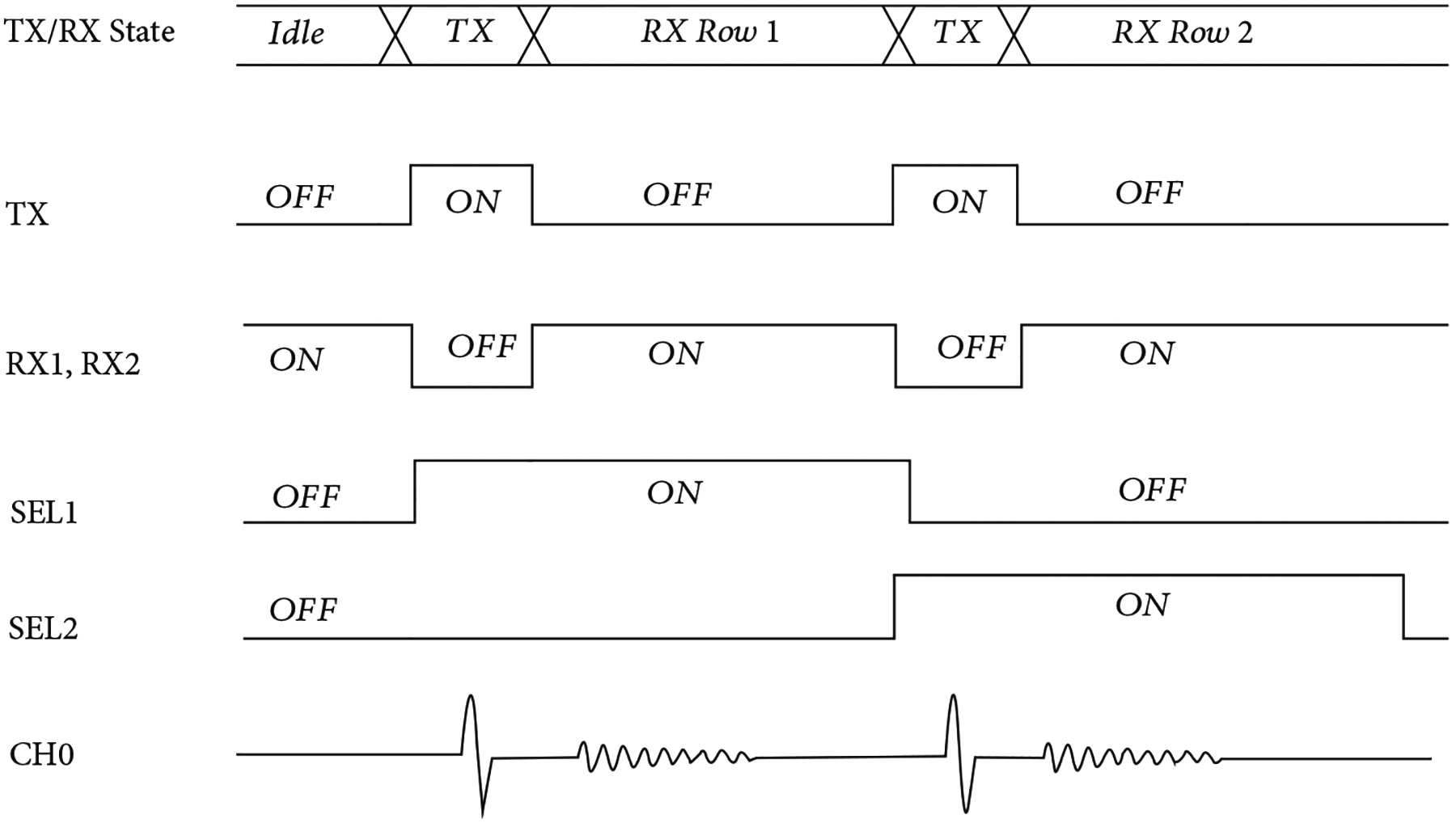
Timing diagram for operation of the unit cell circuitry of Figure 4. The circuit operates in separate TX and RX states, with the TX switch being enabled during the TX state and the two RX switches being enabled during the RX state. This effectively serves to route high voltage signals around the amplifier while also removing the low impedance feedback path of the TX switch during the RX state.

The first-generation unit cell (Figure 4(a)) operates according to the timing diagram shown in Figure 5, and the overall behavior can be better understood with reference to Figures 4(e)–4(g). During the transmit cycle (Tx), the TX switch is turned on, and the RX1 and RX2 switches are turned off, protecting the amplifier. The SEL switch is turned on if the respective unit cell is transmitting (e.g., SEL1 and SEL2) and turned off if the cell is not selected. After the Tx cycle completes, the TX switch is turned off, and the RX1 and RX2 switches are turned on. This connects the transducer (XDCR) to the preamplifier through the RX1 switch. The output of the preamplifier drives the analog bus line in the respective column (CH0) of the unit cell, accessed through the series resistance of the RX2 and SEL switches. The second-generation unit cells have no on-chip buffer at every element, and therefore they do not require the on-chip T/R switches. The commercial off-chip buffers (MAX4805) have their own integrated T/R switches for protection.

The design resistance of the TX and RX1 switches is 500 *Ω* each, whereas the RX2 and SEL switches are each designed to be 250 *Ω*. The feedback network of the first-generation unit cell preamplifier is a 2.5 pF capacitor in parallel with a 100 k*Ω* resistor. Important overall ASIC parameters for the first- and second-generation devices are summarized in Table 1.

As compared to commercial off-the-shelf switching ASICs for ultrasound [41, 43], the on resistance of the switches used in this design is 10–20 times higher (e.g., 1 k*Ω* vs. 50 *Ω*). In addition, the parasitic capacitance of the designed devices is significantly reduced compared to commercial devices (e.g., 10 pF vs. 100 pF). These resistance and capacitance values match more closely the impedance of 2D and 1.75D transducer elements which have on the order of 10–20 times smaller area than the 1D elements for which the commercial devices are intended. As stated previously above, the switches have 10× lower impedance than that of the transducer elements themselves. Even though the switches are higher impedance than typically expected in ultrasound system electronics that interface to large elements at the end of a long cable, they are still low enough to not significantly load the transducer elements during the receive cycle. For the transmit cycle, the high impedance switches are located on the other side of the cable capacitance from the system. The system transmitters do not drive the high cable load capacitance (e.g., 100 pF-150 pF) through the high impedance switches, and therefore the rise time of the transmit pulse is not significantly affected by the RC time constant of the cable capacitance and switch resistance. The switch resistance does play a role when transmitting to the array elements; however, this RC time constant is 10× lower because the element impedance is much higher than the cable impedance.

The SEL switch in the unit cell is controlled by a single data bit that is shifted into the cell and stored in a local flip-flop (D). The flip-flops in the eight cells in each column in the array are tied together in series and form an eight-bit shift register that is used to transfer the data for the switches in the column. The data is shifted into the array at the start of the Tx cycle and also at the start of the Rx cycle. This allows the array to be configured to multiplex different elements on Tx vs. Rx which is important for implementing a full synthetic aperture scanning operation.

### 2.3. Unit Cell Layouts.

**Figure 6:**
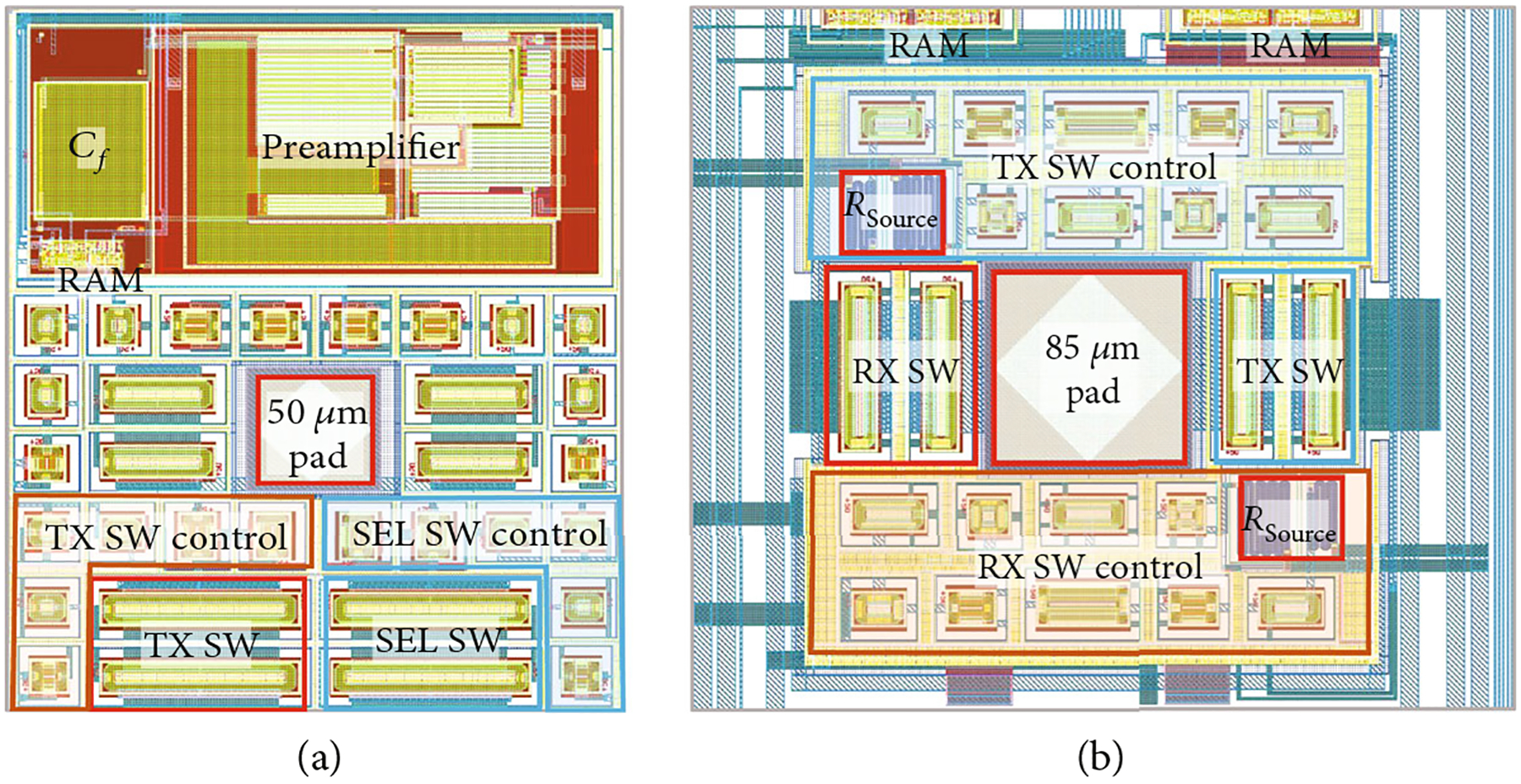
ASIC unit cell layouts: (a) first-generation device and (b) second-generation device.

The layouts of the first- and second-generation unit cells are provided in Figure 6 for comparison. The cells are similar in area, with the first-generation cell (Figure 6(a)) being 325 *μ*m × 275 *μ*m and the second-generation cell (Figure 6(b)) being 327 *μ*m × 382 *μ*m. The first-generation device was designed for direct assembly [33] which necessitated it to be pitch-matched with a 4.5 MHz acoustic array (~300 *μ*m). The second-generation devices did not have this constraint, and therefore their unit cells are designed with less dense layout. This is visible for example at the left and right side of the unit cell layout in Figure 6(b) where there is vertical signal and power supply routing (wide blue lines) which is not seen in Figure 6(a). In fact, the lines are still present in the first-generation devices, but they are routed above the active devices on higher metal layers. This layout design choice was carefully routed to avoid crosstalk and high voltage breakdown; however, without the strict pitch-matching constraint, the second-generation layout simply expanded the area for each unit cell. Another obvious difference between the two layouts is the size of the center transducer assembly pad: 50 *μ*m for first-generation and 85 *μ*m for the second-generation. With reduced requirements for pitch-matching area reduction, it was felt that a more standard pad size would be preferred for wire-bonding and potential flip-chip assembly. One interesting difference between the two layouts is the presence of the low voltage preamplifier in the first-generation devices (Figure 6(a) top). While it consumes a third of the layout of this cell, it is easy to see that when layout constraints are relaxed as with the second-generation cell (Figure 6(b)), the other circuitry readily expands to fill in the extra allowed space. Also, while the first-generation cell has 4 HV switch circuits with only a single SEL switch, the second-generation has only 2 HV switching circuits, and both of these are used for the two selection banks (discussed above). Finally, as can be seen in Figure 6(b), the individual source resistors of the second-generation design consume minimal area and therefore do not constitute an appreciable area penalty for implementation of low charge-injection switching (Section 2.4). In fact, every ASIC design is an optimization of many different often-conflicting parameters including area, cost, power consumption, noise, and operating voltage, among others. The final optimized design area is highly dependent on these specific design choices, as well as the FET sizing design procedure using the process-specific device models and process corners which is a critical step in the final optimization.

### 2.4. Basic Operation of the Unit Switch.

**Figure 7:**
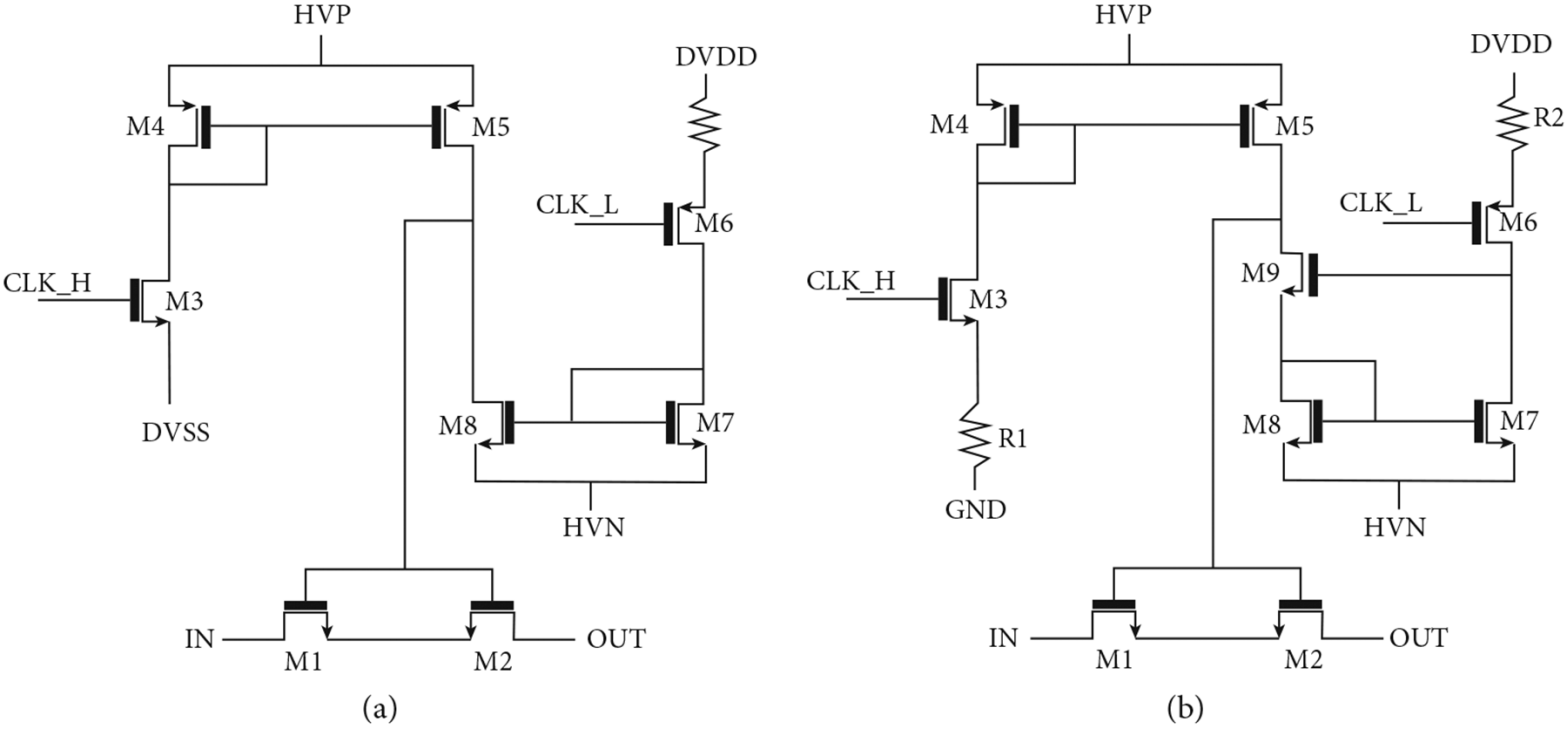
Switch architecture for the first- and second-generation switches. All switches used in each respective unit cell (e.g., select and transmit/receive) utilize these basic architectures. The second-generation switches have source resistors R1 and R2 which reduce the current in the mirrors and thereby slow down charging of the switch device gate capacitances (M1/M2) to limit charge-injection. (b) adapted from [31].

All of the switches in the unit cells of the first- and second-generation ASICs are designed according to the same basic architecture illustrated in Figure 7(a) which is derived from earlier work [42]. The second-generation switches (Figure 7(b)) modify the original architecture to reduce charge-injection as discussed in Section 2.4.4; however, basic operation is very similar to that described in this section. As discussed in this section, the switches operate in STATIC and DYNAMIC modes, with the STATIC mode consuming more current but providing complete control of the gate voltage and the DYNAMIC mode allowing the gate to float up to high voltages which are required for the switch to pass the large (e.g., >5 V) ultrasound transmit signals.

**Figure 8:**
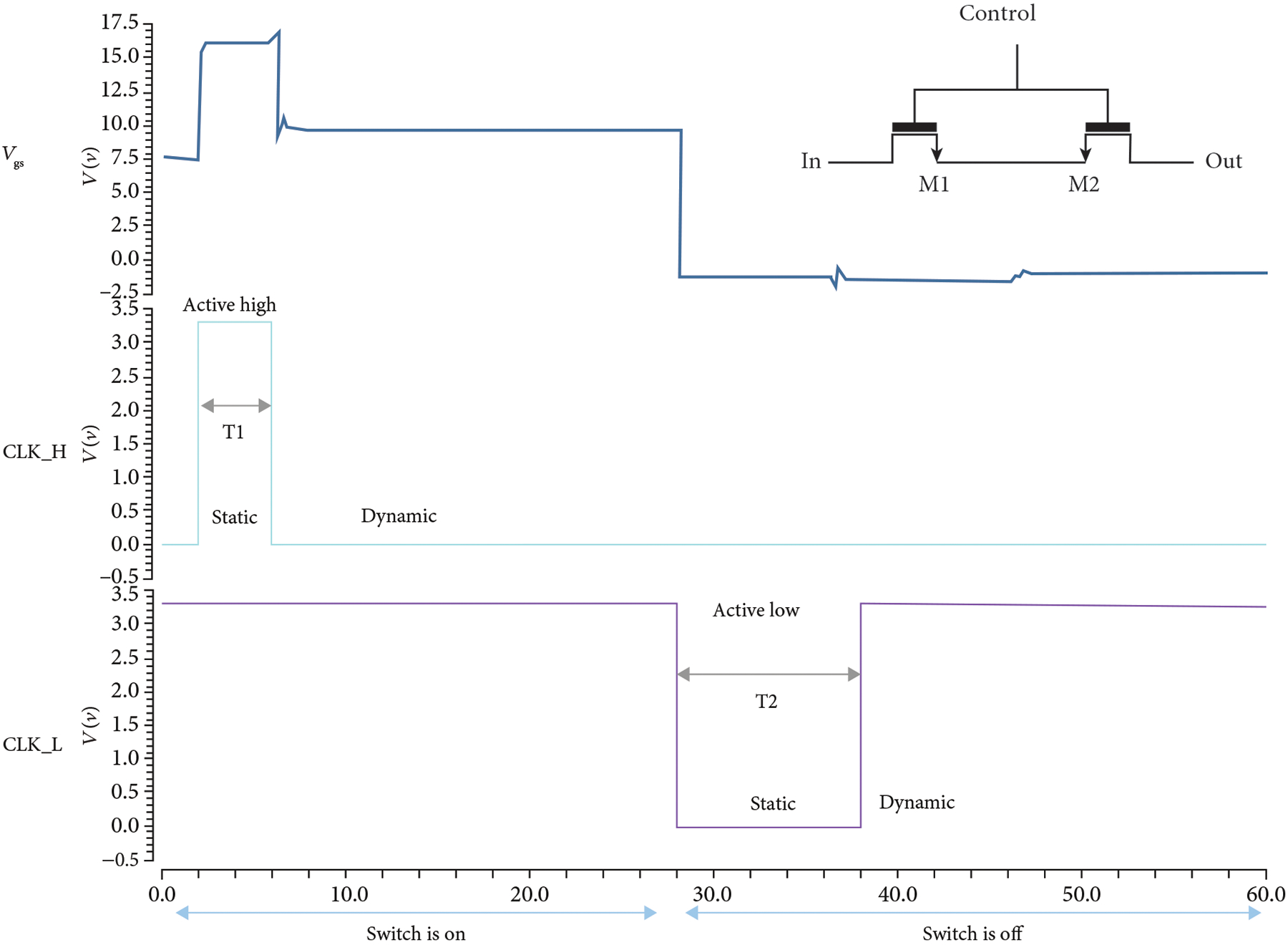
Simulation results for the unit switch. The switch operates in DYNAMIC and STATIC modes: In the STATIC precharge mode, the gates of M1/M2 are charged to a low voltage to turn them ON. Then, in the DYNAMIC mode, the gates float which allow the switch to conserve power and enables high voltage signals to pass through the devices M1 and M2 without damaging their sensitive low voltage gates.

The switch itself is composed of two back-to-back high voltage (HV) lateral DNMOS devices (M1, M2). Two devices are required to block the flow of the current across the parasitic substrate diodes in the DNMOS device structure, thereby providing off-isolation for bipolar signals [7, 42, 44, 45]. The switch is controlled by two level shifters: A high side branch (M3-M5) pulls the gate of the switch up to +HVP in response to a logic input at CLK_H. A low side branch (M6-M8) pulls the gate of the switch down to −HVN in response to a logic input at CLK_L. The nominal high voltage supplies are +/−20 V, and the logic operates between 0 V and +3.3 V. The switch is operated in static and dynamic modes with up to 40 Vpp (+/−20 V) signals with 20 V tolerant thick gate devices. The switch was simulated using Cadence SPECTRE (Cadence Design Systems Inc., San Jose, CA) with results in Figure 8.

#### 2.4.1. Static Mode.

In the static mode (Figure 8), the switch is turned on by applying CLK_H =3.3 V which drives current into the high side current mirror (M4-M5). The current mirror then drives the current to the control node which charges up the parasitic gate capacitance across the shared V_gs_ of the switch devices. In this mode, the logic signal at CLK_H is held high for the entire duration of the period of operation. For example, for the TX1 switch, CLK_H can be held high for the entire Tx cycle (e.g., 10–20 us). The switch is turned off by applying CLK_L =0 V (active low), which drives the current into the low side current mirror (M7-M8), which in turn pulls current out of the control node and discharges the parasitic gate capacitance of the switches, turning them off. In the static mode, CLK_L is held low for the entire duration of the off period. For example, during the Rx cycle, TX1 can be held off by holding CLK_L low for the entire cycle. Operation in the static mode is limited due to the requirement that V_gs_ not exceed the maximum for the selected transistors which therefore requires the transmit signal to be less than 20 V_pp_. In addition, operation in static mode implies continuous current draw which can lead to excess power dissipation.

#### 2.4.2. Dynamic Mode.

To turn the switch on in dynamic mode, CLK_H = 3.3 V is applied for a charging period T1. This charges up the parasitic gate-source capacitance of the switch devices to V_gs_ (ON) = +HV. At the end of the charging period, CLK_H = 0 V, and the current mirror devices are turned off which stops the flow of current into the gate-source capacitance and also allows the control node to float, similar in operation to DRAM [7, 46, 47]. In practice, leakage currents at the gates of the switch devices will discharge the gate capacitance over time and charge-injection causes V_gs_ to decrease slightly (Figure 8).

To turn off the switch, CLK_L = 0 V is applied for a period T2. This discharges the gate-source capacitance of the switches through the low side current mirror until V_gs_ (OFF) = 0 V. V_g_ drops from the high voltage until it reaches the same voltage as V_s_. After that, V_g_ and V_s_ continue to drop together until they both reach −HV, while the low side mirror is operating. When CLK_L returns to +3.3 V, V_g_ and V_s_ both migrate up to the analog ground voltage (+1.65 V), and V_gs_ (OFF) = 0V is again maintained indefinitely.

Operation in the static mode vs. the dynamic mode each have advantages and disadvantages. An important consideration for the array is power dissipation. When CLK_H is turned on, the current mirror draws a static current which can be significant for a large array of tiled modules each comprising a number of switching ASICs. To mitigate this problem as well as to enable operation with bipolar signals greater than the FET V_gs_ level (20 Vpp), the switches can be operated in dynamic mode. During the Tx cycle, off-isolation is critical to prevent crosstalk between elements, and it can be improved by using the switches in the static mode instead of in the dynamic mode during Tx.

#### 2.4.3. Precharge/Hold.

To control the switches, the logic bit DOUT from the flip-flop (D in Figure 4) in each unit cell is routed to a logic block that gates two global control signals for each switch: SSWH and SSWL (not shown). The CLK_H signal (Figures 7 and 8) is generated by the logical combination of DOUT and SSWH, while the CLK_L signal (Figures 7 and 8) is generated by the logical combination of DOUT and SSWL. In this way, the logic bit DOUT in fact does not turn the switch on or off, but instead provides access to the selected switch so that it can be turned on or off using the global control signals. This control architecture necessitates a global precharge and hold operation. During the precharge phase, all the flip-flops are turned on by toggling the global set signal SN. This allows all SEL switches in the array to be turned on using the global SSWH signal. Data are then shifted into the registers in each column with only the switches to be turned off being selected. Then, the global SSWL signal is used to turn off these selected switches. SSWH can then be held for the entire duration of the given cycle as needed to improve off-isolation of the switches. For imaging close to the skin line, it is important to be able to complete the data programming and switch configuration for RX within 1–2 *μ*s. This is accomplished using a high-speed data clock (40 MHz) as well as switch charge-on time of 0.5 *μ*s.

#### 2.4.4. Low Charge-Injection Switching.

The first-generation ASICs displayed significant charge-injection which when coupled into the impedance of the transducer elements manifests itself as a voltage mode “glitch” that can be readily seen on time domain oscilloscope measurements (see Section 3.2.4). These glitches appear when the switches are actuated (turned on or off), and they generate analogous acoustic energy at every element. Due to the fact that these acoustic glitches are all generated at the same time, they create a plane wave which propagates in the medium and can lead to significant (and detrimental) visual artifacts (Section 3.4). Therefore, it was important to resolve this issue, and the second-generation ASICs (Figure 7(b)) were designed with this purpose.

Charge-injection is a well-known phenomenon in general switching circuits [48–50], and there are a number of proposed techniques for mitigating its effects [48, 49]. These include the use of dummy switches to simultaneously emit similar and opposite cancelling charge to offset the charge from the main switches [48], as well as techniques to reduce the edge rate of the control signals at the gates of the switching devices [49]. Figure 7(b) (adapted from [31]) illustrates the topology of the modified switch architecture. The main difference between this circuit and the original switch of Figure 7(a) is the presence of the resistors R1 and R2 in the sources of the switch control MOSFETs M3 and M6. The action of these resistors is to greatly reduce the amount of current that flows in the drains of M5 and M8 when those FETs are turned on which in turn leads to an elongated turn on of the switch. The greatly reduced current in M3/M6 and M4/M7 leads to a greatly reduced mirrored current in M5 and M9, and this results in much slower turn-on of the switch FETs M1 and M2. This is due to the fact that there is much less current to charge up the shared gate capacitance of these switching devices. This elongated turn on has two important benefits: First, as has been documented in general MOSFET based switching circuits [49], the use of a slow turn-on waveform at the FET gates allows the charge time to equilibrate and thereby reduces the net amount of charge that is pushed out from (or pulled into) the drains of the devices. Secondly, and very specifically to ultrasound, slowing the turn-on of the switches shifts the energy in the glitches to lower frequencies where it is effectively filtered by the bandwidth of the transducers themselves. As will be seen in Section 3.2.4, the net result is that the acoustic glitch is suppressed by the low response of the transducers at lower frequencies. The combined effect leads to significant reduction in glitch energy such that the artifacts are significantly reduced in images with the second-generation devices.

The second-generation ASICs (Figures 3(c) and 3(d), adapted from [40]) implement an identical 5 column ×8 row matrix of unit cells as with the first-generation ASICs and are implemented in the same 0.35 *μ*m 50 V CMOS process (AMS H35) [29]. Digital control and actuation of the second-generation switches is very similar to the first with the exception that the slow gate charging to reduce injection means that the switches require more time to turn on and this is programmed into the FPGA control sequence. Another important difference with the second-generation ASICs is the lack of buffer preamplifiers at each element. The design moves the preamplifiers to each column which leads to a significant reduction in power consumption of the entire array.

### 2.5. First- and Second-Generation Receiving Circuit Performance Modeling.

As was illustrated in Figure 4, the first-generation ASIC unit cell integrates high voltage switches along with a respective preamplifier for each array element. The second-generation cells have no preamplifier on-chip and instead use an off-chip commercial buffer (MAX4805). The preamplifier in the first-generation devices is implemented as an inverting amplifier using a standard cell op-amp from the AMS H35 analog library [29]. The details of the circuitry for the op-amp are not described here since the design is a standard cell that is proprietary to AMS. Important parameters of the unit cell are listed Table 2.

The use of an op-amp in the inverting configuration is a common architecture for sensor interface and is also implemented in interface ASICs for ultrasound [10, 38, 51]. Here, we use a capacitive feedback element for gain along with a feedback resistor for DC stabilization. A small signal model of the first-generation unit cell receiving circuitry (Figure 4 (b)) along with a simplified model of the transducer element near resonance was used to derive the overall receive transfer function of the first-generation devices, *H1(s)*, which is given by

(1) $$H1(s)=\frac{sC_{3}R_{1}}{\left(1+sC_{3}R_{4})(1+sC_{1}R_{1}\right)}\frac{R_{3}}{R_{3}+(1+sC_{2}R_{3})R_{2}}\cdot \frac{\omega_{t}}{\omega_{t}+s[1+(sC_{3}R_{1}/(1+s\;C_{3}R_{4})(1+sC_{1}R_{1}))]}.$$

Similarly, a model for the second-generation devices (Figure 4(d), assuming a perfect voltage buffer) is given by

(2) $$H2(s)=\frac{sC_{x}R_{6}}{\left(sC_{x}R_{6}+sC_{x}R_{7}+1\right)}\frac{1}{\left(1+sC_{2}R_{5}\right)}.$$

The definition and values for each of the circuit components in the small signal models of (1) and (2) are listed in Table 2 and correspond to the schematics of Figures 4(b) and 4(d), respectively. The analysis assumes a simple model of the transducer in receive mode as a 16pF capacitance in series with the small signal input voltage. For the case of the second-generation circuit, modeled in (2), there is also a 100 pF series capacitance used in the MAX4805 buffer amplifier circuit that is added to the 16 pF of the elements to yield the 116 pF receive capacitance, C_x_. For the first-generation devices, the signal output is taken after the series resistance of the preamp protection switch (RX2 in Figure 4) and the cell selection switch (SEL in Figure 4). In the second-generation devices, the MAX4805 preamp is assumed to be an ideal voltage mode buffer with input resistance, R6, and output source impedance, R5. The switch on resistance, R7, for the second-generation model (2) is assumed to be 330 *Ω*. The output is dropped across the parallel circuit of the cable load capacitance (C1) and the Verasonics input resistance (R3). The bandwidth of the amplifier is *ω*_t_ = 2*π*f, where *f* is assumed to be 50 MHz for the model, based on the datasheets for the op-amp standard cell library component.

**Figure 9:**
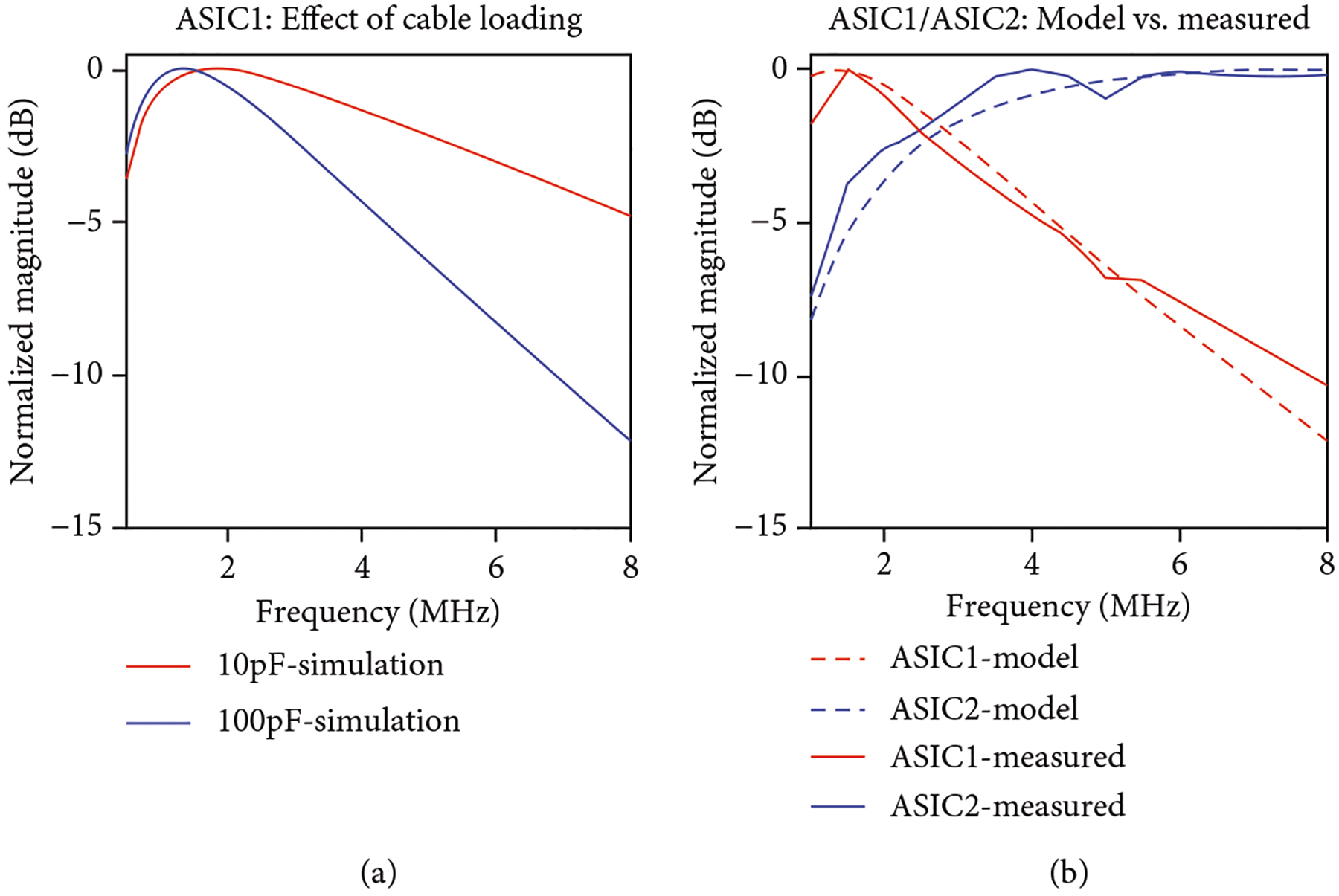
Receive frequency response of the unit cells, (a) model (Equation (1)) comparison of first-generation response when loaded with ultrasound cable (100 pF case) vs. unloaded (10 pF case) as with a PCB connection, and (b) plots of the models of Equation (1) and Equation (2) as compared to actual measured data with the first- and second-generation ASIC modules utilizing a 16 pF series capacitance and function generator as a source load model of the transducers in receive mode.

The normalized magnitude of the frequency response for (1) is plotted in Figure 9(a) for the two cases of assumed cable capacitance load (blue line) and no cable load (red line). For both cases, the analytical model predicts the maximum gain occurring near 1.5 MHz for this first-generation ASIC receive model. For the cable loading condition, the signal then decreases linearly in dB to a −3 dB receive frequency of 3.4 MHz. When operated without a modeled cable load, the −3 dB response instead is extended out to 6 MHz. The operating range for the array modules is between 2.5 MHz and 6 MHz, and within this range, the signal roll-off of the preamplifier is acceptable when not loaded by a cable. The model in (1) however predicts that the signal bandwidth for the first-generation ASICs will be reduced when loaded by the system cable which is in fact what was observed in lab measurements.

Figure 9(b) compares the lab-measured results for the first- and second-generation unit cells interfaced to the Verasonics system with a 16 pF capacitor and a sinewave input modeling the transducer in receive mode. The measured results (solid) are plotted against the modeled results (dashed lines) for both (1) and (2). Here, it is apparent that the second-generation ASIC modules benefit from a wider bandwidth as opposed to the first-generation ASICs. The main reason for this is the loading of the first-generation output SEL switch resistance driving the system cable capacitance. This effect is a consequence of the first-generation architecture in which every unit cell has its own preamplifier, and these are all isolated from the common signal bus by respective select switches. The bandwidth of the circuit could be increased by the addition of another buffer at the top of each column to isolate the SEL switch resistance from the cable load capacitance. This would be a hybrid of Figure 2(a) and Figure 2(b) and would be readily implemented by combining the first-generation ASICs with the MAX4805 buffer with integrated high voltage T/R switch. As discussed above, for smaller elements (e.g., 2D and *λ*/2 pitch), this hybrid topology would be beneficial from a signal bandwidth and sensitivity perspective; however, it comes with an increased power dissipation as noted earlier. In the present 1.75D arrays, we have chosen to utilize the architecture of the second-generation ASICs with a single off-chip buffer for each system channel as in Figure 2(b).

### 2.6. Integration with Ultrasound System.

The first- and second-generation ASICs were integrated with transducers [30, 32, 33] to create transducer/ASIC modules which were then interfaced to a Verasonics Vantage system (Verasonics, Kirkland, WA) which allows for direct control of the beamforming parameters for imaging. The control of the ASICs was accomplished using an FPGA (Xilinx, San Jose, CA) integrated on the distal end of the probe cable, local to the ASICs and transducer array.

The Verasonics system is designed to program industry standard muxed probes using an 8-bit serial interface integrated in the ultrasound cable. To increase flexibility in control of the ASICs, we have implemented a command control protocol on top of this serial interface using codes which serve as keys to a look-up table stored locally in the FPGA and read out in real-time during imaging. To reduce jitter, we used Verasonics system channel 1 as a timing signal that initiates and synchronizes the probe Tx/Rx cycle to the system. The databus rate for the Verasonics communication is 1 MBPS, whereas communication with the ASICs occurs at 40 MPBS. The higher data rate allows reconfiguration of the switches to be accomplished immediately after the end of the transmit cycle so that a minimal amount of the receive signal is lost when different transmit and receive configurations are used (e.g., for synthetic aperture imaging). The FPGA control circuitry also generates all of the timing control signals which are required to actuate the high voltage switches.

## 3. Test Results

### 3.1. Acoustic/Electric Module Fabrication and Validation.

**Figure 10:**
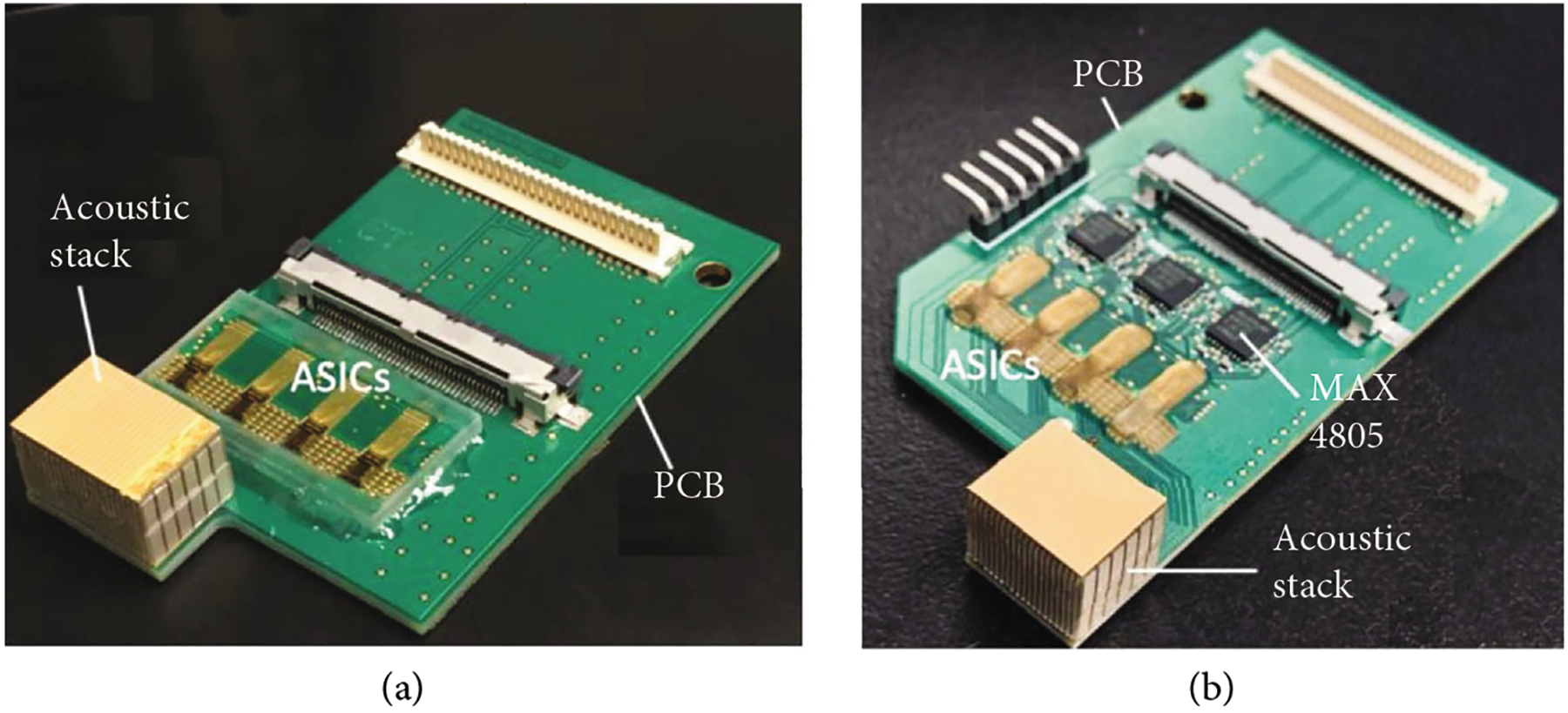
Integration of first- and second-generation ASICs with respective acoustic stacks. (a) first-generation module, (b) second-generation module including MAX4805 receive buffers ((a) adapted from [30] (b) from [31]).

First- and second-generation ASICs and acoustic modules fabricated as described previously [30, 31] were integrated with custom-designed printed circuit boards (Figure 10, adapted from [30, 31]). The ASICs were wire-bonded to the PCB by a vendor, and the acoustic stacks were integrated using a conductive adhesive “stamping” technique described previously [30, 33]. Figure 10(a) shows the first-generation module with the ASICs with on-chip buffering, while Figure 10(b) shows the second-generation modules with the low-charge-injection switch ASICs and similar acoustic stacks. Due to the lack of on-chip buffers in the second-generation ASICs, the modules incorporate three MAX4805 devices (Maxim Integrated, San Jose, CA) for buffering. Both the first- and second-generation ASICs were designed with 8 unit cells in elevation to match with an expected 8 rows in the acoustic matrix; however, due to limitations of the USC acoustic array fabrication, only 6 acoustic rows were used in elevation. In this case, the top and bottom rows in each ASIC column were left unconnected and instead were used for electrical testing. It should also be noted that the USC acoustic array fabrication process standardized on 20 elements per module in azimuth. As this is not a power of 2, we used 3 MAX4805 parts (with 8 buffer channels each) for the second-generation modules to yield 24 total transmit/receive channels, of which 20 were used for acoustic element connections, and the remaining 4 channels per module were used for electrical testing and evaluation during development.

Prior to integrating the ASICs with the acoustic stack PCBs, ASIC functionality was evaluated using a needle probe and benchtop testing. The ASICs were connected to power and ground, as well as individual analog channel input/outputs. The ASICs were also directly interfaced to the FPGA controller which generated all of the timing and data signals for programming the ASIC and actuating the switches in real-time. A probe needle landed on the transducer interface pad at the center of each first-generation ASIC unit cell and provided direct access for monitoring the multiplexed signals as well as for applying test signals for input stimulus for the preamplifiers.

**Figure 11:**
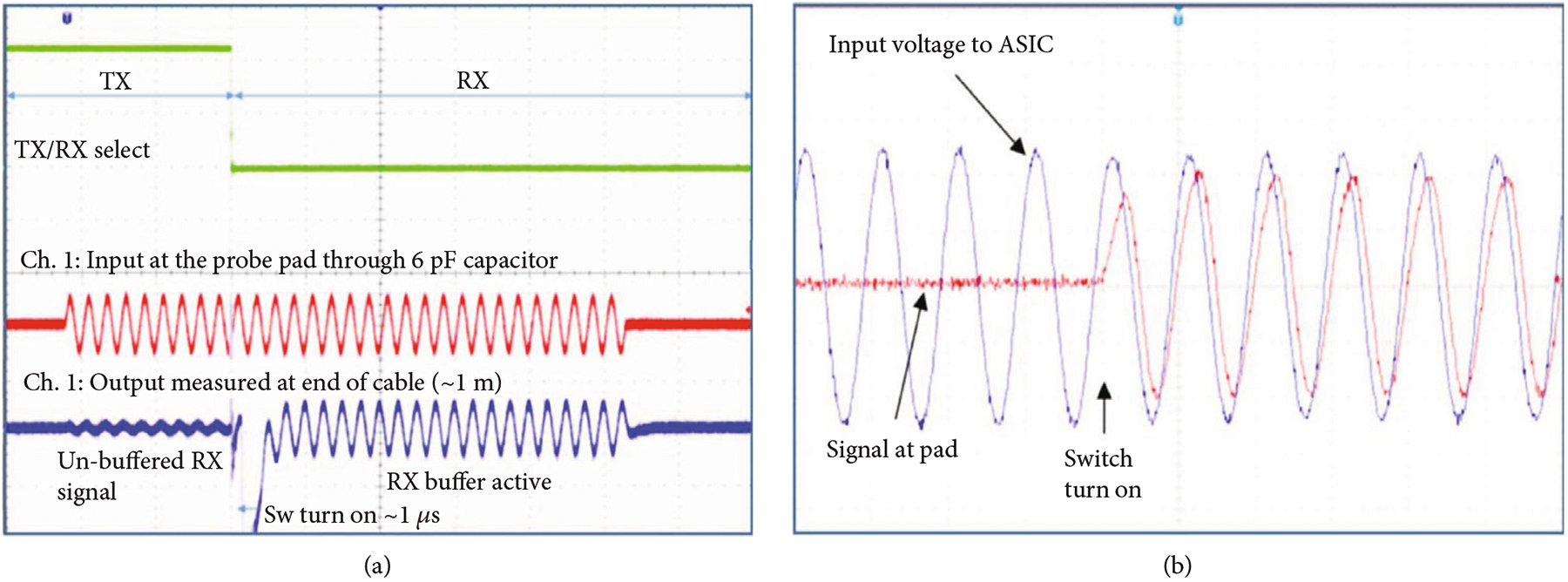
Validation of electrical function of the first-generation ASICs. (a) receive test and (b) high voltage switch test.

We validated both the transmit and receive functionality separately as shown in Figure 11. Figure 11(a) shows the receive path test, with a low voltage signal applied at the probe needle landing on the transducer interface pad. The green trace in the figure is the control signal for actuating the TX, RX1, and RX2 switches to switch the input signal between the bypass path around the amplifier and the amplifier itself. The red trace is the low voltage analog signal applied through the capacitance to the probe needle. The blue trace is the measured signal at the output of the ASIC preamplifier, as provided to the oscilloscope through one of the I/O pads at the top of the ASIC column for the respective cell. This test demonstrates low voltage functionality of the switching circuits as well as amplification through the first-generation ASIC receive preamplifier.

To validate the high voltage switch functionality as shown in Figures 11(a) and 11(b), a high voltage test signal (blue) was applied to the I/O pads at the top of the ASIC, which then routed this test signal down the analog bus in the respective column of the ASIC. The digital control signals were actuated to turn on the high voltage switches in the cells, and the resulting transmitted signal (red) was detected at the probe needle. After the switch is digitally actuated, the red trace begins to follow the blue trace demonstrating effective operation of the device. The switch series resistance (900 *Ω*) combined with the cable load capacitance leads to some propagation delay and signal attenuation as seen in Figure 11(b) when comparing the blue and red traces.

### 3.2. Characterization of Performance Parameters.

The first- and second-generation modules were interfaced to a Verasonics Vantage system which was used to further characterize the performance of these devices.

In particular, the system was used to determine the input parasitic capacitance of the two different modules which is an important determinant of parasitic loading on the transducer and affects the signal response during the receive cycle. As discussed in the introduction, parasitic capacitances are also a potential issue when shorting together multiple switch channels for parallel multiplexing of elements. The transmit on resistance of the multiplexing paths in the two ASIC module architectures was also assessed using the Verasonics system. Finally, the voltage magnitude for the glitches as well as off-isolation were measured. Each of these is discussed in this section.

#### 3.2.1. Parasitic Capacitance.

**Figure 12:**
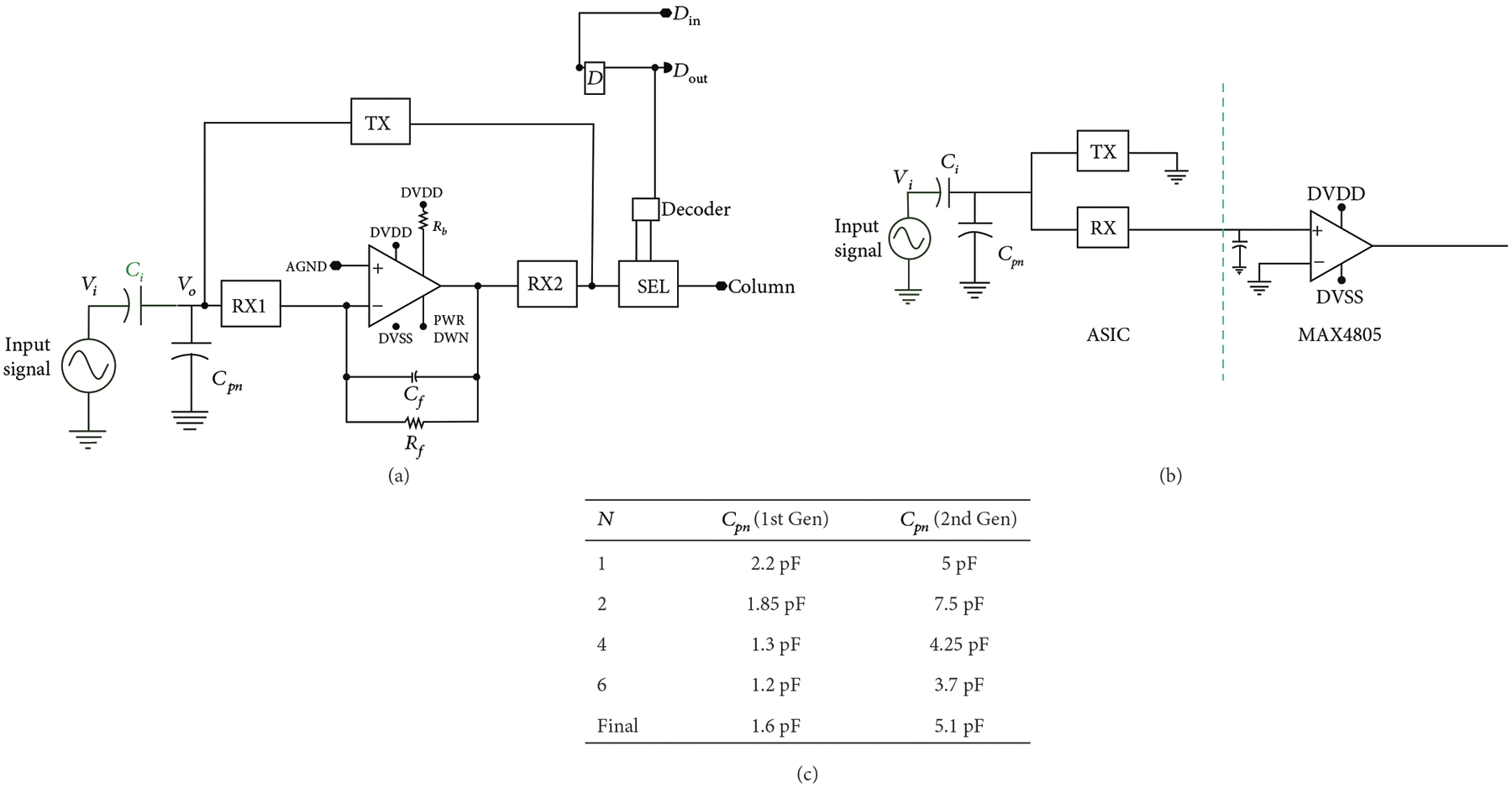
The parasitic capacitances at the inputs of the first- and second-generation modules were determined using the circuits in (a) for first- and (b) for second-generation devices. The table in (c) shows calculated values for first- and second-generation with increasing *N*. The final value for Cpn was calculated as the mean of the 4 measured values.

The parasitic capacitances at the transducer inputs of the first- and second-generation modules were determined as shown in Figure 12. The circuit in Figure 12(a) was used for the first-generation module and Figure 12(b) for the second-generation, where a test signal of 100 mV_pp_ at 3 MHz was coupled through a test capacitance (C_i_ = 16 pF) to the input capacitance C_pn_. The voltage Vo at the module input was measured using a high impedance FET probe (C4121, Caltest Electronics). The voltage divider of C_pn_ and C_i_ yields an attenuation factor *α* which can then be used to compute C_i_ from

(3) $$\alpha =\frac{\text{Ci}}{\text{Ci}+\text{NCpn}},$$

The load capacitance of the FET probe (2.6 pF) was taken into account. Multiple elements, *N*, were successively shorted together to create an increasingly larger aggregate capacitance which thereby reduces the effect of any stray capacitance on the measurements. The table in (c) shows calculated values for first- and second-generation, with an increasing *N*. The final value for Cpn in each case was calculated as the mean of the 4 measured values.

#### 3.2.2. Transmit Mux on Resistance.

**Figure 13:**
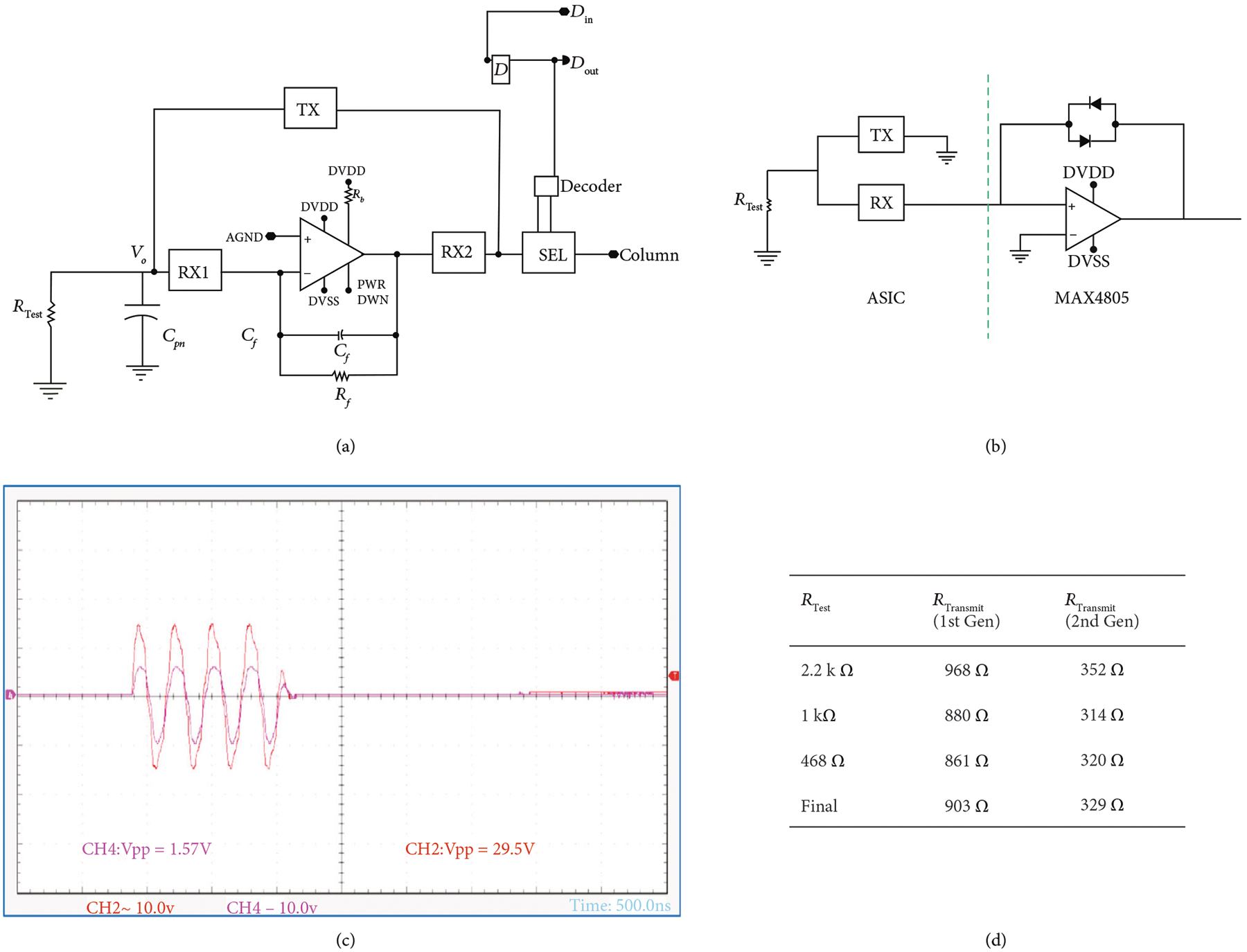
Transmit ON resistance of the individual ASIC module channels was measured as illustrated in (a) for the first-generation architecture and (b) for the second-generation device. The attenuation, *α*, is visible in (c) where the red trace is the input signal and the purple trace is the attenuated voltage measured across R_Test_. Calculated R_Transmit_ values for first- and second-generation are tabulated in (d).

The transmit ON resistance of the individual ASIC module channels was measured as illustrated in Figure 13. Figures 13(a) and 13(b) show the circuits used for the first- and second-generation ASICs, respectively. Three calibrated discrete wire-wound test resistances, R_Test_, with values of 1 k*Ω*, 468 *Ω*, and 2.2 k*Ω* were used in turn. A four cycle transmit voltage at 3.5 MHz and 30 V_pp_ was transmitted from the Verasonics system to the ASIC module. A voltage divider formed by the ASIC module series resistance and the off-module load of R_Test_ attenuates the measured transmit voltage. The voltage V_o_ at the transducer connection for the channel was measured across R_Test_ using a high-impedance FET probe (CT4121, Caltest Electronics). The attenuation, *α*, is visible in Figure 13(c) where the red trace is the input signal and the purple trace is the attenuated voltage measured across R_Test_. The value of R_Transmit_ can then be determined from

(4) $$\alpha =\frac{R_{\text{Test}}}{R_{\text{Test}}+R_{\text{Transmit}}}.$$

The final value for R_Transmit_ was determined as the average of the three measured values for both the first- and second-generation as shown in the Table (d). The reduced on resistance for the second-generation devices was due to the use of large high voltage switch devices in the new design. In addition, the second-generation devices have only a single series switch, whereas the first-generation devices have two switches in series (SEL + TX) which increases the total series switch resistance. As was discussed above in Section 2.5, the lower source impedance of the second-generation unit cells improves the sensitivity and bandwidth due to a reduction in the effect of the coupled load impedances (e.g., parasitics on the PCB and cable).

#### 3.2.3. Transmit Off-Isolation.

**Figure 14:**
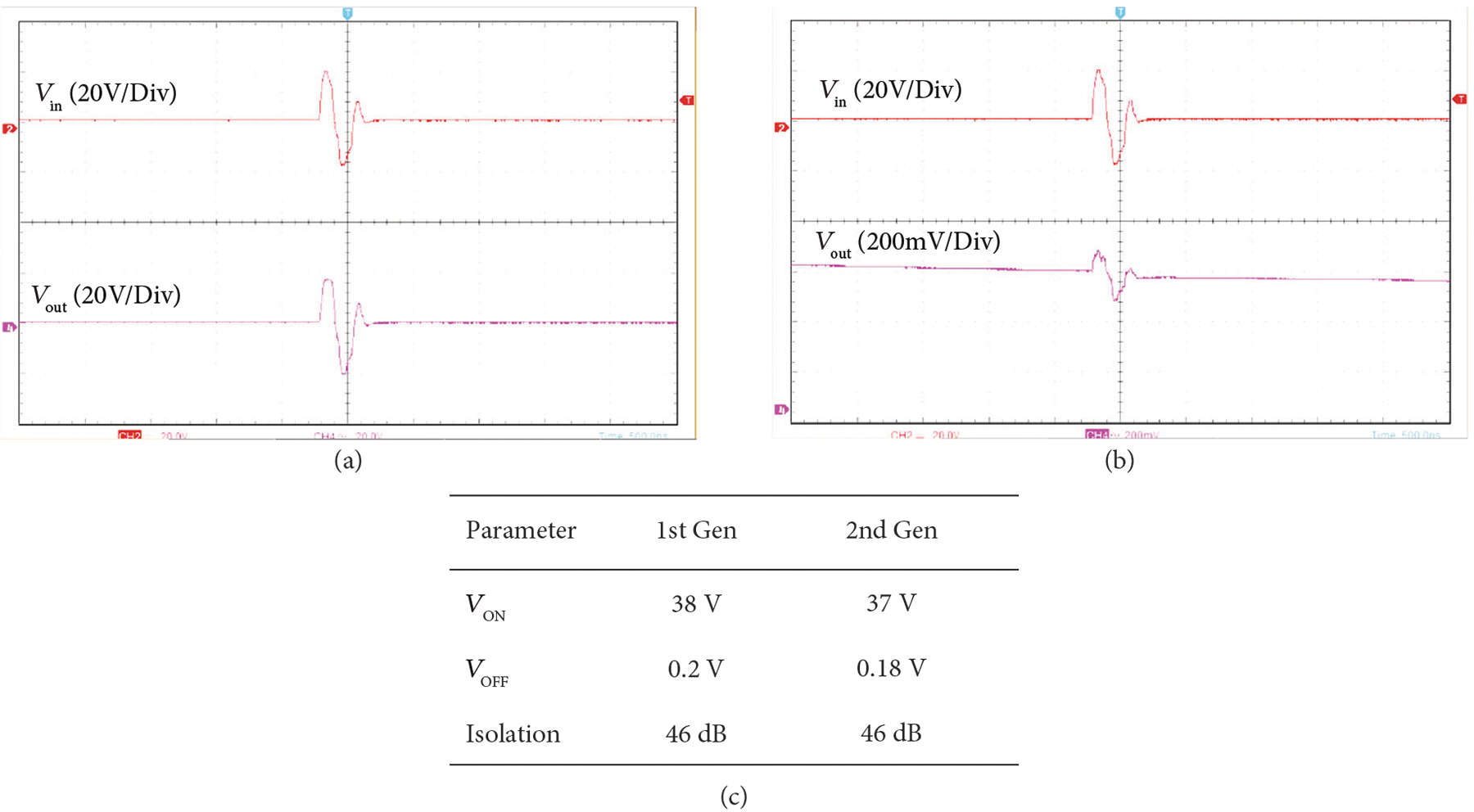
The transmit off-isolation was measured by operating the first- and second-generation modules interfaced to the Verasonics system with a single cycle pulse at 38 Vpp and Fc = 3.5 MHz. (a) shows the result when the switch is ON, and (b) shows the result when the switch is OFF. As can be seen in (b), the transmitted pulse is effectively reduced by 46 dB with the switch turned off, and (c) tabulates these results.

The transmit off-isolation was measured by operating the first- and second-generation modules interfaced to the Verasonics system with a single cycle pulse at 38V_pp_ and Fc = 3:5 MHz. The output of the module transmit path was measured using a high impedance FET probe (CT4121, Caltest Electronics) so as not to load the resulting off-isolation signal. Figures 14(a) and 14(b) show the representative measurements of the input and output voltage waveforms for the first-generation module. The results were similar for the second-generation (not shown). The transmit pulse observed in Figure 14(a) at the output is also the maximum transmit voltage that the architecture will support with +/−20 V supplies. Figure 14(a) shows the result when the switch is ON, whereas Figure 14(b) shows the result when the switch is OFF. As can be seen in Figure 14(b), the transmitted pulse is effectively reduced by 46 dB with the switch turned off.

#### 3.2.4. Glitch Level Measurements.

**Figure 15:**
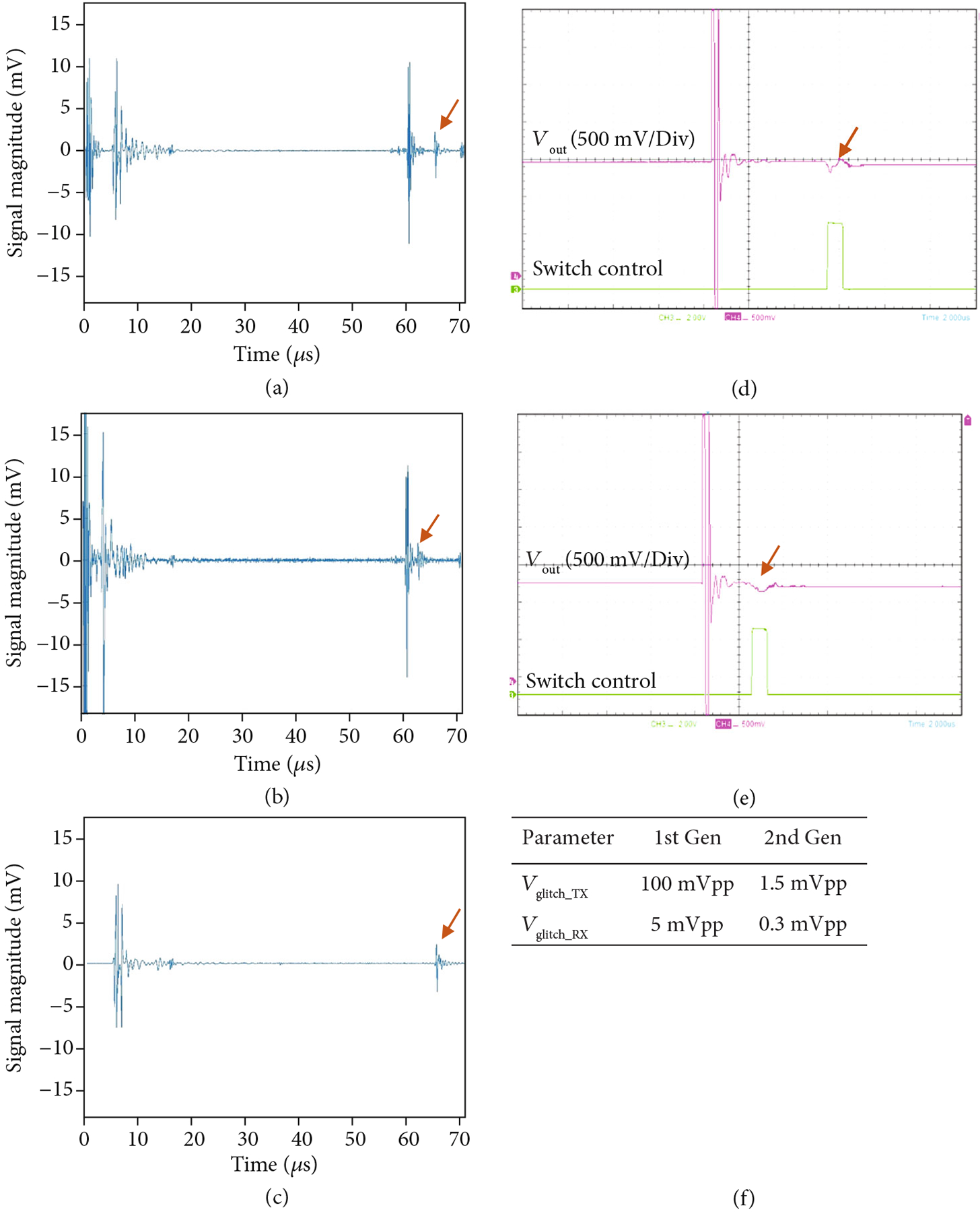
Measurement of Glitch level. (a) Verasonics receive signal with arrow indicating the glitch echo, (d) analogous voltage oscilloscope trace measurement, with arrow indicating observed outgoing glitch waveform (pink trace) in response to digital control signal (green trace) that turns on the switch. Translating control signal (e) moves the glitch response (arrow in (b)). (c) When transmit pulses are turned off, the acoustic response prior to the glitch disappears; however, the glitch itself is unchanged indicating that it is caused by switching transients.

The results of glitch level measurement experiments are shown in Figure 15. The glitch level was measured by operating the first- and second-generation ASIC modules with the Verasonics system and observing the resulting glitch level in the receive signal of the Verasonics system and also at the transducer connection of the modules using a high impedance FET probe (CT4121, Caltest Electronics). The modules were loaded using a gel phantom that was 50 mm thick, and a 50 mm thick quartz block was situated at the bottom of the phantom and acted as the reflecting target. The Verasonics receive signal is shown in Figure 15(a) with an arrow indicating the glitch echo. The large echo before that is the actual acoustic response of the front face of the target. An analogous voltage oscilloscope trace measurement is shown in Figure 15(d), with the arrow indicating the observed outgoing glitch waveform (pink trace) in response to the digital control signal (green trace) that turns on the switch. By moving the control signal (e), we observe analogous movement of the glitch response (arrow in b). When the transmit pulses are turned off (c), the acoustic response prior to the glitch disappears; however, the glitch itself is unchanged. This is a strong indication that the glitches are caused by switching transients as the switch actuation on the ASIC modules occurs regardless of the output level of the Verasonics transmitters. The absolute transmitted voltage level of the glitches for both the first- and second-generation ASIC device modules was measured using the oscilloscope and is tabulated in Figure 15(f). We also measured the received glitch voltage levels at the Verasonics system channels, and these are also tabulated in Figure 15(f).

The results of the measurements in this section have been summarized in Table 3. They are compared to parameters as listed in datasheets for industry standard multiplexing and preamplifier devices as well as another similar custom ASIC device [42].

### 3.3. Ultrasonic Phantom Imaging with the First- and Second-Generation ASIC Modules.

**Figure 16:**
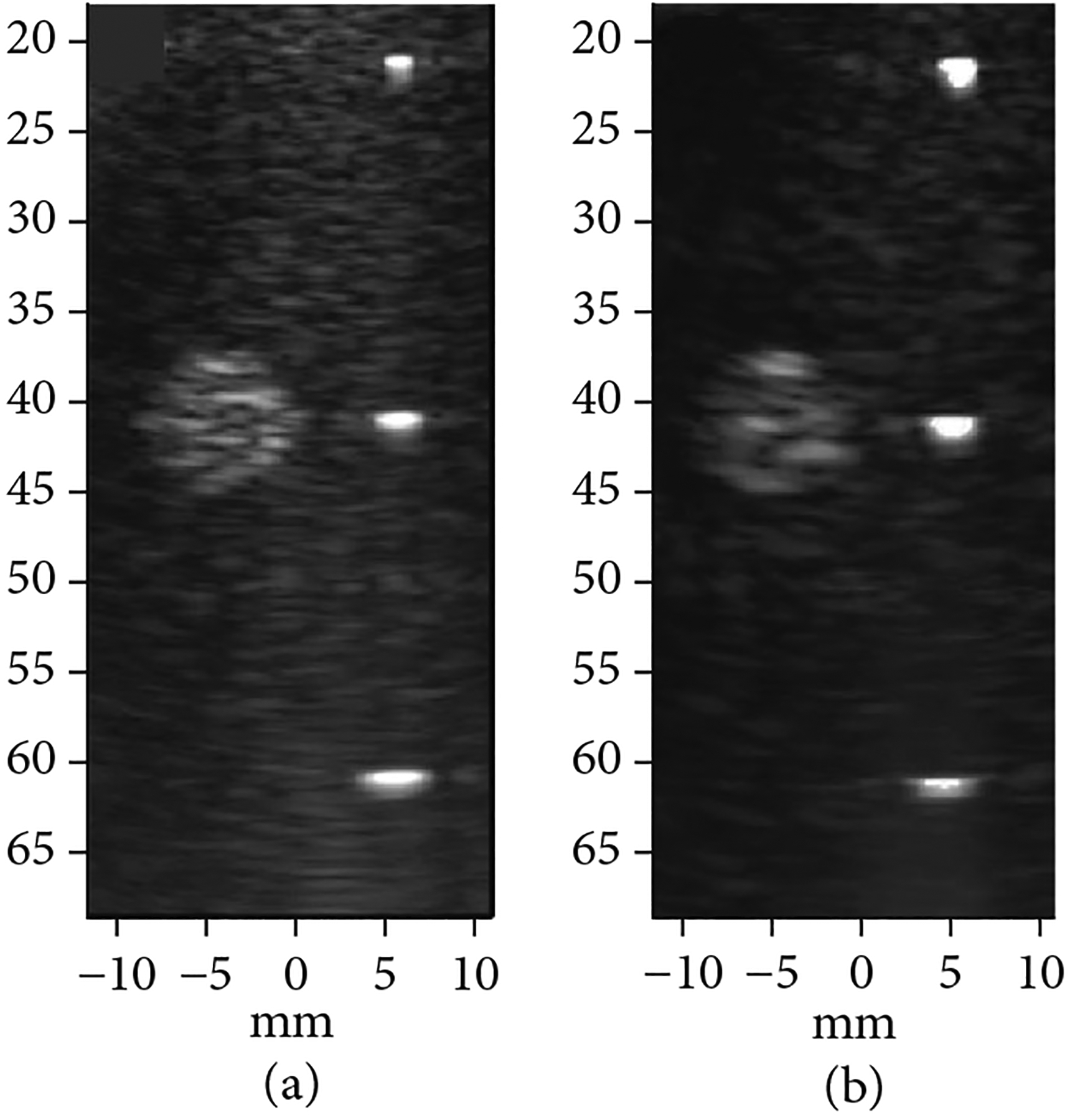
Acoustic imaging tests for the (a) first- and (b) second-generation acoustic/ASIC modules imaging a highly echogenic cyst and a vertical group of wires in a CIRS 054GS ultrasound phantom. Displayed dynamic range for the images was 40 dB.

The first- and second-generation modules were used to image 100 *μ*m nylon wire targets and a hyperechoic cyst in a CIRS 054GS acoustic phantom to compare the imaging performance of the two architectures (Figure 16). Detailed pulse-echo testing of the two acoustic stacks prior to assembly showed that the second-generation stacks had a lower bandwidth as compared to the first-generation stacks (65% vs. 82%) which was due to sub-dicing and over-lapping the first matching layer of the acoustic elements in the second-generation stacks. This reduced acoustic element fractional bandwidth is completely unrelated to the ASIC electronics architecture and has no effect on the glitches; however, it did result in a decreased axial resolution seen by comparing Figures 16(a) and 16 (b). We have since demonstrated >80% fractional bandwidth with second-generation ASICs integrated with a different acoustic array [40]. Both the first- and second-generation ASICs displayed low lateral resolution as compared to production probes due to the small apertures of the test modules (only 20 elements in azimuth compared with 128 for production probes). The goal of the project is to tile multiple of the validated acoustic/electric modules together to build a large aperture which should demonstrate excellent lateral resolution due to the inverse dependence of image resolution on array aperture width (see Figure 1).

### 3.4. Mitigation of Charge-Injection Induced Image Artifact with New Switch Architecture.

As described previously, the first-generation ASIC switch architecture was observed to generate significant glitches which lead to image artifacts. These artifacts are especially important in a large area array due to the fact that each switching operation produces simultaneous emission of glitch energy into the medium on all the elements that are connected to switches that are being actuated. With a large array, the very large number of such elements results in a plane wave being coupled into the medium. When this plane wave is incident on bright reflectors (e.g., the leading edge of a large air-filled structure such as lung), it has the potential to create a structure in the resulting beamformed images that is not actually present in the imaged tissue. Such anomalous imaging artifacts are critical in ultrasonic imaging due to the fact that they represent confounding information for the sonographer and radiologist that has the potential to lead to erroneous conclusions and false-positive assessments. Therefore, such imaging artifacts must be mitigated, and this is the purpose of the second-generation ASIC with low charge-injection switching.

**Figure 17:**
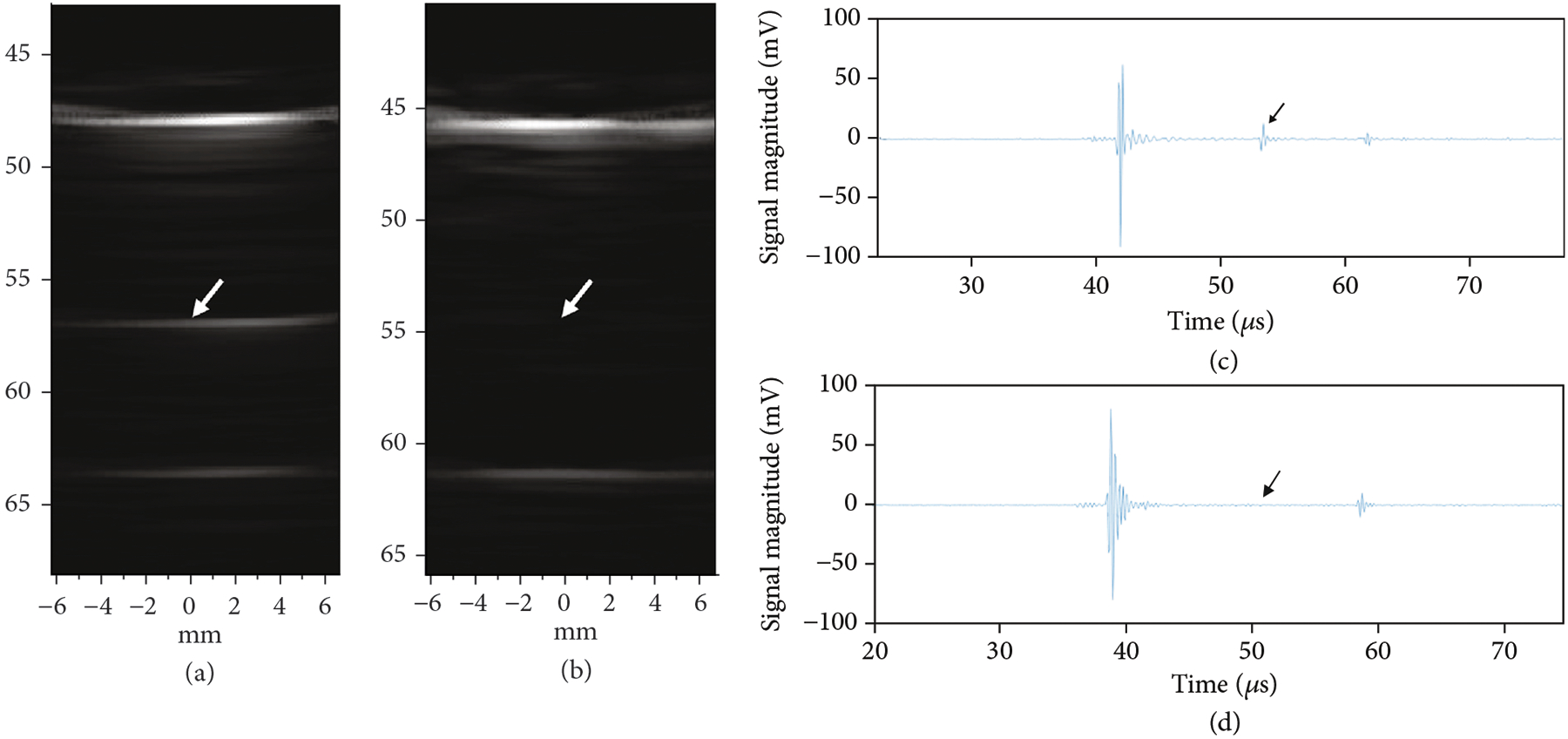
Glitch reduction in second-generation devices when imaging quartz target, (a) first-generation ASIC module (b) second generation ASIC module, arrows show the location of the image artifact due to glitches (c-d) show plots of the Verasonics RF receive data for (c) the first-generation and (d) the second generation ASIC mdoules, arrows indicate where the glitches are located. (adapted from [31]).

Figure 17 provides a comparison of imaging with the first- and second-generation ASIC device modules illustrating the effects of the glitch reduction. The test imaging setup (not shown) consisted of the respective ASIC modules with integrated transducer array, a 45 mm gel phantom standoff, as well as a 58 mm thick quartz target to create the echoes in the image. Figure 17(a) shows imaging with the first-generation ASIC module where the strong echo line near 48 mm depth is the main response off of the surface of the quartz target. Further below that, at 57 mm depth, the echo from the outgoing glitch pulse is visible. At a depth of 64 mm, the echo from the back face of the quartz target is found. (Note that the speed of sound in quartz is faster than in water and this accounts for the shortened expected depth of the block in the image.) The second echo (white arrow in Figure 17(a)) is generated by the same surface in the target as the first (i.e., the front face of the quartz block), and therefore it is an unwanted image artifact.

Figure 17(b) shows imaging with the second-generation ASIC module with the improved glitch reducing switch architecture. Note the lack of an image artifact between the front and rear face echoes. Arrows in the images in (a) and (b) show the location of the image artifact due to echoes created by the glitches being transmitted into the medium when the ASIC switch configuration was changed to the receive mode. Figure 17(c) and 17(d) show the time domain plots of the Verasonics RF receive data for the first- and second-generation ASIC modules, respectively. The arrows indicate where the glitches are located in the RF data, again demonstrating the ability of the new architecture to effectively suppress the glitch.

## 4. Discussion

Throughout this paper, we have sought to highlight important differences between the first- and second-generation devices and will summarize some of these comparisons here in this section. It is important first to note that as in Figure 2, an important tradeoff exists between integrating buffer amplifiers at every element (Figure 2(a)) and integrating them instead at every column and system channel (Figure 2(b)). This tradeoff decision depends greatly on the size of the acoustic elements and their proportional impedance which needs to be properly isolated from the loading of the system cable and receive electronics. The two different ASIC generations demonstrate different aspects of this tradeoff of localized amplification. Specifically, the first-generation ASICs have buffering at each element which better matches the 2D element impedance to the cable loading. The second-generation devices have buffering only at each column which was a compromise to reduce power at the expense of some increased loading of the elements. We also note that the second-generation modules demonstrated lower bandwidth due to the lower fractional bandwidth of the acoustic elements themselves. This effect was observed during testing of the bare elements prior to assembly to the ASIC modules. We have built new electronics modules using the second-generation ASICs with acoustic elements produced by a commercial vendor which demonstrated >80% fractional bandwidth [40].

Another very important difference between the two ASIC architectures is the presence and mitigation of glitch-related switching artifacts. The first-generation switching devices which demonstrate a significant charge-injection–related switching glitch are representative of many switch architectures in use today, and in particular, historically utilized commercial switch devices have much larger injected charge. Due to the fact that the output signal of the elements is proportional to the charging current driving the devices, the use of such large switches results in very significant glitch energy for small 2D and 1.75D array elements, and this effect is only made worse as the number of switched elements grows. Therefore, the practical realization of very large arrays of switched elements relies on switch devices with very low injected charge glitches. The second-generation devices provide significant reduction of the glitches using a reduction in the drain current of the charging devices. As opposed to techniques that utilize dummy switches to cancel the spurious injected charge, the use of reduced gate charging current does not rely on precise matching of MOSFET devices and is therefore more robust to differences in layout.

There are a number of ASIC based devices reported in the literature which have utilized unipolar pulsers which save valuable ASIC area [50, 51]. The HV switches described here are optimized for bipolar pulsing, and our ASICs do not implement pulsers locally; these are instead integrated in the Verasonics system. In our intended application, bipolar excitation is required for high quality imaging with harmonics because unipolar pulses have significant harmonic components which are transmitted into the tissue and confound the harmonics from oscillating bubbles or the tissue itself.

Matching has historically been an important technique for improving the sensitivity of linear array probes and typically involves the use of inductors or coils placed either at the distal or proximal end of the cable depending on the particular method of signal optimization. With the integration of active electronics at the distal end of the cable closely integrated with the acoustic elements themselves, matching becomes a question of isolating and properly buffering the high impedance elements from the loading of the cable. Matching of elements on transmit is done by sizing the MOSFET switches to be lower on resistance than the impedance of the elements. The maximum current is delivered as long as the driver impedance is less than the load because it will always be limited by the load impedance. On receive in both ASIC architectures presented here, we have integrated buffer amplifiers which have high impedance inputs with low impedance outputs to effectively drive the low impedance load of the cable. This is a well-known technique for improving receive sensitivity for high impedance 2D elements [8–10, 38].

A very important trade-off with probe-integrated electronics is that the series resistance of the high voltage switches can be made larger than the typical ultrasound system (e.g., 50 *Ω*) due to the fact that the small 2D elements themselves have high impedance. This fact has made possible very high-density and high functionality switching matrix implementations [16, 33, 38, 42, 47, 50] which were not previously possible when very large low resistance high voltages switches were used. R_in_ in the Verasonics can be set to a higher level which is one way to attempt to help match the system electronics to the high impedance elements. Unfortunately, with the standard cable, this does not affect the loading that the elements see due to the cable itself which can be as much as 100 pF-300 pF (180 Ohms at 3 MHz). Therefore, locally integrated buffer amplifiers with low output driving impedance are required, and we have implemented these in both the first- and second-generation devices.

Finally, loading of the elements by the cable as well as the series output resistance of the switched buffer in the first-generation ASICs caused a roll-off at higher frequencies which is clearly demonstrated in Figure 8. The model in Equation (1) provides a realistic simulation of the behavior of the system as demonstrated in agreement to the measured data. In the second generation, the buffer amplifiers are no longer positioned behind the high resistance MOSFET switches and instead talk directly to the cable. This leads to improved performance at high frequencies as can be seen in Figure 9(b) for the second-generation ASIC’s measured data.

## 5. Conclusions

Large aperture ultrasonic arrays implemented by tiling multiple pretested modules together hold potential for improved image resolution and depth of penetration in a number of important imaging applications for ultrasound. These include imaging for cancer detection in the breast and liver, as well as therapy applications with HIFU. With this work, we have presented the detailed design and analysis of first- and second-generation ASICs and ASIC modules intended to be tiled to form large array apertures through a modular array approach. In particular, such large aperture modular arrays can be used to implement 1.75D type imaging apertures that benefit from improved slice thickness that rejects out of plane artifacts. The resulting increase in contrast to noise (CNR) is critical for effective differentiation of fluid filled cysts from cancerous ones. The developed high voltage ASICs were implemented using 0.35 *μ*m high voltage (50 V) CMOS and integrated with high-density 1.75D transducer arrays implemented using wide-bandwidth 1–3 composites of PIN-PMN-PT material and a 3D printed interposer backing for high-density interface. Presented imaging, electrical, and acoustic test results demonstrate functioning ASICs and modules. We observed a significant image artifact in the first-generation ASIC modules which was due to a voltage glitch that coupled into the transducers to create a plane wave of acoustic energy in the imaged medium. This artifact was effectively mitigated with the design of the second-generation ASIC switches that have reduced charge-injection at their input and output terminals and demonstrated a reduction of between −20 and −30 dB in observed artifact depending on operating conditions. In our future work, we are integrating larger systems of tiled modules similar to the ones presented here, working towards a fully integrated probe for human imaging trials.

## Figures and Tables

**Table 1:** Design parameters of the first- and second-generation ASICs.

Parameter	1^st^ generation	2^nd^ generation
ASIC width	1.8 mm	1.8 mm
ASIC height	3.6 mm	4.4 mm
Number of rows	8	8
Number of columns	5	5
Unit cell width	340 *μ*m	382 *μ*m
Unit cell height	275 *μ*m	327 *μ*m
Transducer pad	50 *μ*m	85 *μ*m

**Table 2:** Design parameters and final model values for 1^st^ and 2^nd^ generation ASIC unit cell receiving circuits with reference to Figure 4.

Parameter	Model parameter	Model value
Feedback capacitor	C1	2.5 pF
Feedback resistor	R1	100 k*Ω*
TX, RX1 R_ON_	R4	411 *Ω*
RX2 + SEL R_ON_	R2	520 *Ω*
Cable capacitance	C2	100 pF
Verasonics R_in_	R3	8 k*Ω*
Transducer model	C3	6 pF
2^nd^ gen cell receive capacitance	Cx	116 pF
2^nd^ gen buffer source resistance	R5	65 *Ω*
2^nd^ gen buffer input resistance	R6	4 k*Ω*
2^nd^ gen switch ON resistance	R7	330 *Ω*

**Table 3:** Comparison of reported parameters for switching ultrasound ASICs.

Parameter	First-generation module	Second-generation module	[42]	MPS MP4816 [43]	Supertex HV2761 [41]	Maxim MAX4805 [52]
Matrix	5 × 8	5 × 8	64 × 1	16 × 1	24 × 1	8 × 1
ASIC process	0.35 *μ*m	0.35 *μ*m	0.35 *μ*m	—	—	BiCMOS
Max V_TX_	38 Vpp	38 Vpp	50 Vpp	180 Vpp	200 Vpp	200 Vpp
TX Sw Ron	900 Ω	330 *Ω*	180 *Ω*	12.5 Ω	20 Ω	1.5 Ω
Glitch − XDCR	5 mVpp	0.3 mVpp	—	—	—	—
Glitch − 50 Ω	—^3^	—^3^	—	30 mVpp	300 mVpp	—
Off-isolation − XDCR	−46 dB	−46 dB	−17 dB	—	−30 dB	—
Off-isolation − 50 Ω	—	—	—	−66 dB	−60 dB	—
C_parasitic_	1.5 pF	5 pF	—	11 pF	30 pF	3.5 pF
Receive bandwidth	4 MHz	10 MHz	70 MHz	45 MHz	—	44 MHz
Die/package	3.7 mm × 1.8 mm	4.5 mm × 1.9 mm	6 mm × 1.6 mm	7 mm × 7 mm	9 mm × 9 mm	5 mm × 5 mm
Power/channel	77 mW	24 mW^2^	—	63 *μ*W^1^	1.7 mW^1^	8.5 mW

1 OEM switching power calculated for 64 switch events in 32 ms.

2 Current second-generation modules use +/−5 V for preamp supplies as opposed to the manufacturer suggested +/−2 V which makes the power greater than it should be. This will be corrected in future work.

3 ASIC switch parameters for off-isolation and glitch level are not designed for 50 Ω operation and therefore were not measured with 50 Ω load.

## Data Availability

The data used to support this work are available from the corresponding author upon request.
