# Erratum to “Highly Integrated Multiplexing and Buffering Electronics for Large Aperture Ultrasonic Arrays”

**DOI:** 10.34133/2022/9818934

**Published:** 2022-09-27

**Authors:** Robert Wodnicki, Haochen Kang, Di Li, Douglas N. Stephens, Hayong Jung, Yizhe Sun, Ruimin Chen, Lai-Ming Jiang, Nestor E. Cabrera-Munoz, Josquin Foiret, Qifa Zhou, Katherine W. Ferrara

**Affiliations:** ^1^Department of Biomedical Engineering, University of Southern California, Los Angeles, CA, USA; ^2^Department of Biomedical Engineering, University of California, Davis, Davis, CA, USA; ^3^Molecular Imaging Program at Stanford University, Stanford, CAUSA; ^4^USC Roski Eye Institute, University of Southern California, Los Angeles, CA, USA

In the article titled “Highly Integrated Multiplexing and Buffering Electronics for Large Aperture Ultrasonic Arrays” [1], there was an error in figure 7. The figure should show as per the submitted files. The corrected figure is shown below and is listed as Figure 1:

**Figure 1:**
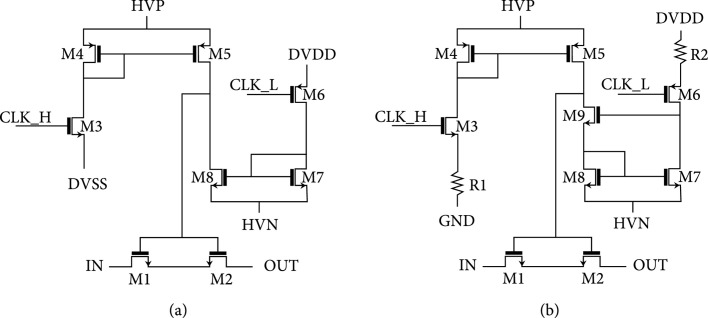
Switch architecture for the first- and second-generation switches. All switches used in each respective unit cell (e.g., select and transmit/receive) utilize these basic architectures. The second-generation switches have source resistors R1 and R2 which reduce the current in the mirrors and thereby slow down charging of the switch device gate capacitances (M1/M2) to limit charge-injection. (b) Adapted from [31].
